# Ethical–Regulatory Guidelines for AI in Palliative Care Rehabilitation

**DOI:** 10.3390/healthcare14070895

**Published:** 2026-03-31

**Authors:** Daniela Oliveira, Sofia B. Nunes, Francisca Rego, Rui Nunes

**Affiliations:** 1 Faculty of Medicine, University of Porto, 4200-319 Porto, Portugal; asnunes@med.up.pt (S.B.N.); mfrego@med.up.pt (F.R.); ruinunes@med.up.pt (R.N.); 2 RISE-Health, Faculty of Medicine, University of Porto, Alameda Prof. Hernâni Monteiro, 4200-319 Porto, Portugal

**Keywords:** AI governance, artificial intelligence, clinical decision support, ethical–regulatory guidance, palliative care rehabilitation, patient-centred care

## Abstract

**Highlights:**

**What are the main findings?**

- This study identifies five core ethical–regulatory domains guiding the responsible use of AI in palliative care rehabilitation.
- High-level international AI ethics frameworks require contextual translation to address the specific vulnerabilities and relational dimensions of palliative rehabilitation settings.

**What are the implications of the main findings?**

- The proposed ethical–regulatory domains support clinically meaningful, proportionate, and human-centred integration of AI in rehabilitation decision-making.
- The findings provide structured guidance to inform governance, institutional policy development, and regulatory oversight in ethically sensitive healthcare contexts.

**Abstract:**

Background/Objectives: The integration of artificial intelligence (AI) into rehabilitation practice has expanded rapidly, including its emerging application in palliative care contexts. Although international organisations have established ethical and governance frameworks for AI in healthcare, these initiatives remain largely high-level and are not specifically tailored to the clinical complexity, vulnerability, and relational dimensions of palliative care rehabilitation. The absence of context-specific ethical–regulatory guidance poses challenges for responsible implementation in ethically sensitive settings. This study aimed to consolidate ethically grounded regulatory guidance for the use of AI in palliative care rehabilitation by translating existing international principles into context-sensitive domains. Methods: A qualitative documentary analysis with a normative ethical–regulatory orientation was conducted using the READ (Ready, Extract, Analyse, Distil) framework. Authoritative international policy, governance, and regulatory documents addressing AI in healthcare were identified and analysed. Data were extracted using a structured analytical table and coded according to predefined ethical–regulatory domains derived from previously published ethical guidelines and verified through documentary analysis. Results: The analysis identified five convergent ethical–regulatory domains recurrent across international governance frameworks: (1) Human oversight and clinical responsibility; (2) Patient autonomy, preferences, and proportionality; (3) Transparency and explainability; (4) Fairness, equity, and non-discrimination; and (5) Professional competence and ethical literacy. These domains were synthesised into practical ethical–regulatory considerations linking ethical principles with governance expectations and clinical implementation requirements. Conclusions: This study provides context-sensitive ethical–regulatory guidance that bridges high-level AI governance principles with the operational realities of palliative care rehabilitation. By systematising and operationalising existing ethical norms, the proposed framework supports responsible clinical decision-making, strengthens institutional accountability, and safeguards patient dignity and autonomy in vulnerable care contexts.

## 1. Introduction

The integration of artificial intelligence (AI) into healthcare systems has accelerated rapidly in recent years, encompassing a wide range of clinical domains, including diagnosis, decision support, monitoring, and personalised interventions. Advances in machine learning, data analytics, and digital health infrastructures have enabled AI-driven systems to support clinical decision-making and tailor interventions to individual patient characteristics [1,2].

In rehabilitation, AI applications are increasingly used to support exercise prescription, functional assessment, remote monitoring, and adaptive therapy planning, particularly through wearable devices, intelligent assistive technologies, and data-driven decision-support tools [3,4].

However, international frameworks such as those proposed by WHO, UNESCO, and the OECD are intentionally cross-sectoral and formulated at a high level of abstraction, offering ethical principles that are not tailored to specific clinical contexts or vulnerable patient populations, such as palliative care patients [5,6,7,8]. In clinically complex settings such as palliative care, the application of generic ethical principles without contextual adaptation may be insufficient to support ethically sound decision-making, particularly where proportionality, relational care, and evolving patient goals are central [9]. Defined as a person-centred and dignity-oriented approach, palliative care prioritises quality of life, relief of suffering, respect for patient autonomy, proportionality of interventions, and the presence of meaningful human relationships throughout the disease trajectory [10,11].

Within this context, patients often experience heightened vulnerability due to advanced illness, fluctuating clinical status, complex symptom burden, and dependence on multidisciplinary care. These characteristics demand careful ethical reflection when introducing digital or automated technologies into clinical decision-making processes.

Rehabilitation is increasingly recognised as an integral component of palliative care, aimed at maintaining functional capacity, supporting autonomy, and enhancing participation in daily life, even in the presence of progressive disease [12,13]. Evidence suggests that tailored exercise and physiotherapy interventions can improve fatigue, functional independence, symptom control, and quality of life in patients receiving palliative care [14,15]. However, it is important to remember that rehabilitation in palliative care is not oriented toward curative goals but toward proportionate, individualised, and ethically grounded interventions that align with patients’ values and preferences throughout the progress of the illness. In this context, proportionality extends beyond clinical risk–benefit assessments to include patients’ subjective tolerance, functional priorities, and evolving preferences over time.

Meanwhile, AI-supported tools are increasingly being explored within rehabilitation practice, including algorithm-assisted exercise planning, adaptive training programmes, and continuous functional monitoring through wearable and digital technologies [3,16]. These systems promise enhanced personalisation, efficiency, and scalability of rehabilitation interventions. However, the introduction of AI into palliative rehabilitation raises distinct ethical concerns, particularly regarding clinical responsibility, preservation of patient autonomy, transparency of algorithmic recommendations, and the risk of over-standardisation in contexts requiring nuanced, value-sensitive judgement. AI systems typically rely on statistical optimisation and pattern generalisation across aggregated datasets. In contrast, palliative rehabilitation requires proportionate and individualised decisions grounded in evolving patient values and relational care contexts.

Despite the proliferation of AI ethics frameworks and regulatory initiatives, there remains no integrated ethical–regulatory model specifically addressing the use of AI in palliative care rehabilitation. Existing frameworks are typically cross-sectoral and articulated at a high level of abstraction, providing general ethical principles without explicit contextual adaptation to ethically sensitive clinical domains [7,17]. Consequently, many current approaches rely on non-binding ethical guidance or broad regulatory standards which, while valuable, are often insufficiently operationalised within concrete clinical governance structures [18,19,20].

A focused search of PubMed, Scopus, and Web of Science using combinations of the terms “artificial intelligence”, “palliative care”, “rehabilitation”, “ethics”, and “governance” did not identify any integrated regulatory-operational frameworks specifically addressing AI-supported rehabilitation in palliative care contexts. While relevant literature exists on AI ethics in healthcare more broadly, on technological innovation in rehabilitation, and on ethical principles in palliative care separately, no structured framework translating international AI governance principles into operational guidance for this specific clinical intersection was identified.

Such guideline-only approaches may be insufficient to address the clinical complexity, vulnerability, and relational dimensions inherent to palliative care, where decision-making is highly contextual and ethically sensitive [21,22]. Moreover, international health governance bodies have highlighted the need to complement ethical principles with governance and regulatory mechanisms to ensure accountability and practical applicability, particularly in high-risk healthcare contexts [5,7].

This ethical and regulatory gap underscores the need for context-specific guidance that aligns high-level AI governance principles with the realities of rehabilitation practice in palliative care. Addressing this gap is essential to support healthcare professionals in using AI tools responsibly, while safeguarding patient dignity, autonomy, and safety.

The authors have previously published ethical guidelines addressing AI-supported exercise prescription in palliative care [23]. Those guidelines were primarily normative in orientation and focused on articulating ethical principles to guide clinical judgement. They did not involve systematic documentary analysis of international governance instruments, nor did they consolidate ethical principles into an explicitly structured ethical–regulatory framework aligned with institutional accountability and regulatory expectations. The present study therefore represents a methodological extension, moving from normative articulation to documentary consolidation and regulatory-operational translation.

In the present study, the term “ethical–regulatory guidance” refers not merely to a set of ethical principles, nor solely to binding legal requirements, but to the structured translation of ethical norms into clinically and institutionally applicable responsibilities. This includes allocation of professional accountability for AI-assisted decisions, documentation and traceability requirements, governance mechanisms, and safeguards ensuring proportional and context-sensitive implementation.

The aim of this study is to consolidate ethically grounded regulatory guidance for the use of artificial intelligence in palliative care rehabilitation. Through a qualitative documentary analysis of international ethical, governance, and regulatory sources, this study articulates existing ethical principles into context-sensitive ethical–regulatory domains. By translating high-level AI governance frameworks into practical guidance applicable to palliative rehabilitation, this work seeks to contribute to the responsible integration of AI technologies into ethically complex clinical settings.

## 2. Materials and Methods

### 2.1. Study Design

This study adopted a normative documentary analysis to examine authoritative international policy, governance, and ethics documents relevant to artificial intelligence in healthcare [24]. This methodological approach was selected to enable structured ethical interpretation and regulatory contextualisation of high-level governance principles within the specific domain of palliative care rehabilitation. The emphasis was placed on qualitative interpretation, ethical coherence, and regulatory applicability, consistent with the study’s aim of producing ethically grounded and practically relevant guidance.

The analytical process was organised according to the READ framework (Ready, Extract, Analyse, Distil), which provides a structured approach to organising and synthesising documentary materials in health policy research [25]. This framework guided document preparation, structured extraction of relevant ethical and regulatory elements, thematic analysis, and consolidation of findings into applied ethical–regulatory domains.

The ethical–regulatory domains applied in this study were informed by previously published ethical guidelines developed by the authors [23] and served as analytical lenses for documentary interpretation. The detailed derivation and consolidation of these domains are described in Section 2.3.

### 2.2. Document Corpus and Identification Process

The documentary corpus consisted of authoritative international policy, governance, and regulatory documents addressing ethical and governance aspects of artificial intelligence (AI) in healthcare.

**Figure 1:**
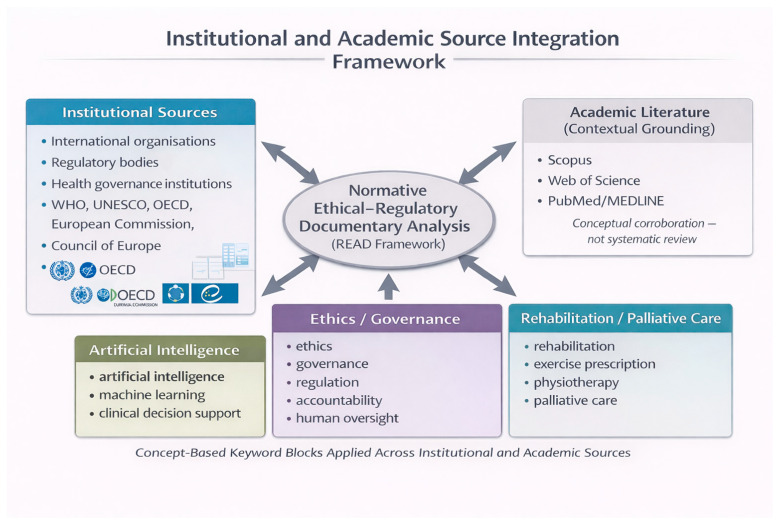
Institutional and academic source integration framework. Authoritative institutional documents constituted the primary documentary corpus for normative ethical–regulatory analysis. Academic database searches were conducted to support contextual grounding and conceptual alignment of ethical–regulatory themes. Concept-based keyword blocks guided document identification across institutional and academic sources. This approach did not constitute a systematic literature review. Instead, it supported structured documentary integration within a normative analytical framework.

A structured identification strategy was adopted, consistent with the normative and policy-oriented objectives of the study. The overall source integration framework is illustrated in Figure 1.

The document identification process focused on internationally recognised organisations and regulatory bodies with established authority in AI governance and health policy. Official institutional websites were consulted directly, including those of the World Health Organization (WHO), UNESCO, the Organisation for Economic Co-operation and Development (OECD), the European Commission, the European Parliament, and related European governance bodies. Internal search functions and document repositories available on these official platforms were used to locate relevant reports, recommendations, regulatory proposals, and adopted legal instruments.

The identification process was guided by the following inclusion criteria:

(i) documents issued within the last 10 years, reflecting contemporary AI governance developments;
(ii) documents formally adopted, endorsed, or issued by recognised international organisations or supranational regulatory authorities;
(iii) documents explicitly addressing ethical, governance, or regulatory aspects of AI applicable to healthcare or clinical decision-making contexts.

Documents were excluded if they:

(i) lacked formal institutional endorsement;
(ii) focused exclusively on technical or engineering aspects without ethical or regulatory discussion;
(iii) represented opinion pieces, commentary articles, or media publications;
(iv) constituted draft or superseded versions where final or updated instruments were available.

Application of these criteria resulted in a final corpus of nine institutional documents forming the primary documentary dataset for analysis. A complete list of the institutional documents included in the analysis, with URLs and dates of access, is provided in Supplementary Material S1.

The selected documents included major international ethical recommendations, governance frameworks, regulatory guidance documents, and legally binding regulatory instruments issued by international and European governance bodies addressing the governance of artificial intelligence in healthcare contexts.

Document identification and screening were conducted by the first author, with uncertainties and borderline cases discussed iteratively with the supervisory research team. This collaborative process supported consistency in document selection and contributed to reducing potential interpretive bias throughout the analytical process.

Explicit reference to palliative care or rehabilitation was not required for inclusion, as the purpose of the analysis was to translate high-level ethical and regulatory principles into the specific context of palliative care rehabilitation.

Academic Literature Contextualisation

In addition to institutional documents, academic databases (Scopus, Web of Science, and PubMed/MEDLINE) were consulted to support conceptual grounding and contextual alignment of ethical–regulatory themes. Searches were conducted using concept-based keyword blocks combining terms related to artificial intelligence, ethics/governance/regulation, and rehabilitation/palliative care.

This academic consultation was not designed as a systematic literature review and did not aim to provide exhaustive coverage of empirical studies. Rather, it served to identify recurring ethical and governance discussions within scholarly literature that were conceptually aligned with the domains identified in the institutional corpus.

Academic sources were used to support interpretation and discussion but did not constitute part of the primary documentary dataset.

The detailed search strategies used for academic database consultation (PubMed/MEDLINE, Scopus, and Web of Science) are provided in Supplementary Material S2.

### 2.3. Ethical–Regulatory Domains and Analytical Framework

As previously described, the ethical–regulatory domains used in this study were deductively derived from previously published ethical guidelines developed by the authors for the use of AI in palliative care rehabilitation. These guidelines were originally grounded in established bioethical principles—namely autonomy, beneficence, non-maleficence, justice, and professional responsibility—and informed by international ethical and governance frameworks addressing AI in healthcare.

Rather than generating new categories inductively from the analysed documents, the present study adopted a deductive analytical approach, structuring existing ethical guidance into a set of ethical–regulatory domains suitable for documentary analysis and regulatory interpretation. The documents were nevertheless examined for additional ethical or regulatory themes that might require refinement of the analytical framework.

No additional domains emerged that required modification of the analytical structure.

This deductive strategy was selected to ensure conceptual continuity between the authors’ prior normative and conceptual work and the present regulatory-oriented analysis. It also reflects recurring themes identified across major international AI governance frameworks, including human oversight, respect for autonomy, transparency, fairness, and professional competence.

Although privacy and data protection are central concerns in AI governance, they were not retained as a standalone domain in the present analysis. This decision reflects the study’s focus on ethical–regulatory dimensions most directly implicated in clinical decision-making and rehabilitation practice in palliative care contexts. Data protection considerations were treated as transversal elements embedded within broader governance structures.

The original eight ethical guidelines previously published by the authors [23] were analytically consolidated into five ethical–regulatory domains to support thematic synthesis and reduce conceptual redundancy. This consolidation process aimed to group conceptually related ethical requirements into stable analytical categories that could be consistently applied across heterogeneous documentary sources.

The resulting domains were: (1) Human Oversight and Clinical Responsibility; (2) Patient Autonomy, Preferences, and Proportionality; (3) Transparency and Explainability; (4) Fairness, Equity, and Non-Discrimination; and (5) Professional Competence and Ethical Literacy.

Each domain represents a distinct yet interrelated ethical–regulatory concern that is particularly important in the context of palliative care rehabilitation.

Palliative care is characterised by heightened patient vulnerability, fluctuating clinical trajectories, and a strong emphasis on dignity, proportionality, and relational care. These characteristics increase the ethical risks associated with inappropriate or uncritical use of AI systems and highlight the need to translate generic AI ethics frameworks into context-sensitive guidance.

Accordingly, the selected domains were not intended to function as abstract ethical principles, but as applied ethical–regulatory lenses through which international governance documents could be interpreted and translated into context-sensitive guidance.

The domains were operationalised as analytical categories within the documentary analysis process. During data extraction and analysis, ethical principles, regulatory expectations, and implementation-related requirements identified in the analysed documents were coded and mapped to the corresponding domain. This structured approach facilitated traceability, transparency, and analytical coherence throughout the process.

By adopting this domain-based analytical framework, the study aimed to bridge high-level AI governance principles with the practical realities of clinical decision-making in palliative care rehabilitation.

The resulting ethical–regulatory domains provided the foundation for synthesising international documentary evidence into actionable guidance that supports responsible, proportionate, and human-centred integration of AI technologies in this ethically sensitive clinical context.

While this deductive approach ensured conceptual continuity, it may introduce a risk of conceptual circularity or confirmation bias. To address this, the analysis remained open to the identification of additional or divergent ethical–regulatory themes emerging from the documentary corpus, allowing the framework to be critically examined against independent international governance sources.

### 2.4. Documentary Analysis Process

Following document identification and selection, all included documents underwent structured data extraction and thematic analysis. A structured data extraction table was developed to ensure transparency and traceability (see Supplementary Material S3). The table supported systematic comparison of ethical principles and governance elements across the analysed documents.

For each document, information was extracted on publication details, document type and geographical scope, stated objectives, key ethical principles, regulatory or governance elements, implementation or operational recommendations, and references to equity or vulnerable populations. In addition, relevant excerpts were recorded to support traceability and analytical transparency.

The extracted information was subsequently coded according to predefined ethical–regulatory domains, which were derived from previously published ethical guidelines developed by the authors and consolidated for analytical purposes.

The domains used for coding were:

(i) Human Oversight and Clinical Responsibility.
(ii) Patient Autonomy, Preferences, and Proportionality.
(iii) Transparency and Explainability.
(iv) Fairness, Equity, and Non-Discrimination.
(v) Professional Competence and Ethical Literacy.

Documents could be coded across multiple domains where relevant, reflecting the cross-cutting nature of ethical and regulatory considerations in artificial intelligence governance. Thematic synthesis was conducted within and across domains, enabling the identification of convergent ethical principles, regulatory expectations, and implementation considerations across the documentary corpus. Attention was given to how high-level ethical and governance principles were articulated, operationalised, or translated into practice within healthcare contexts.

Findings from the thematic analysis were synthesised to support the consolidation of ethical–regulatory guidelines for the use of artificial intelligence in palliative care rehabilitation. This process involved translating recurring principles identified in the documentary corpus into context-specific guidance, explicitly linking ethical principles to regulatory implications and practical clinical requirements.

## 3. Results

### 3.1. Identified Ethical–Regulatory Domains

The documentary analysis identified five convergent ethical–regulatory domains that were consistently observed across the analysed institutional and policy documents and that are particularly relevant when translating high-level AI ethics into ethical–regulatory guidance for palliative care rehabilitation.

The principles informing these domains were consistently identified across multiple types of institutional sources included in the corpus, including international governance frameworks, regulatory instruments, and institutional policy guidance documents addressing the responsible use of artificial intelligence in healthcare (see Supplementary Material S3).

**Figure 2:**
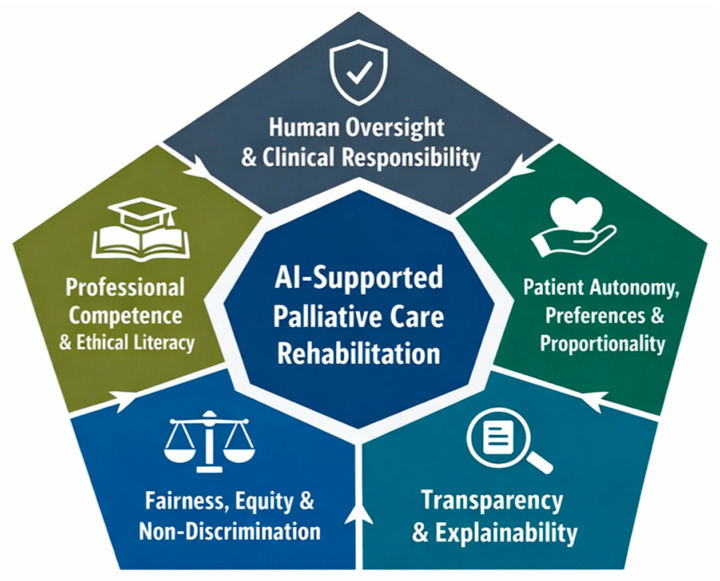
Ethical–regulatory domains guiding the use of artificial intelligence in palliative care rehabilitation. The figure illustrates the five interrelated ethical–regulatory domains identified through documentary analysis. These domains function as a normative framework to ensure that AI-supported rehabilitation in palliative care remains clinically responsible, ethically grounded, and aligned with patient dignity, autonomy, and proportionality.

These domains are: (1) Human Oversight and Clinical Responsibility; (2) Patient Autonomy, Preferences, and Proportionality; (3) Transparency and Explainability; (4) Fairness, Equity, and Non-Discrimination; and (5) Professional Competence and Ethical Literacy (see Figure 2).

Each domain is summarised below in terms of its normative grounding, regulatory references in international instruments, and contextual relevance for palliative rehabilitation.

(1) Human Oversight and Clinical Responsibility

This domain refers to ethical and regulatory requirements emphasising that AI systems should function as decision-support tools rather than autonomous decision-makers. Several analysed institutional documents emphasise human-in-the-loop (or human-on-the-loop) arrangements to ensure traceability, accountability, and the attribution of final clinical responsibility to qualified professionals [5,26].

Within the analysed documents, particular emphasis is placed on maintaining final clinical responsibility with qualified human professionals, especially in complex and high-risk healthcare contexts.

Documents reviewed describe requirements such as documented review of AI outputs, clear escalation pathways when AI and clinician judgements conflict, and institutional governance that assigns roles and audit mechanisms for AI-supported decisions.

These findings are consistent with the authors’ previously published ethical guidelines for AI-supported exercise prescription in palliative care:

- Guideline 1: AI should complement—and never replace—human clinical judgement.
- Guideline 3: There should be constant clinical supervision of the exercises suggested by AI.

(2) Patient Autonomy, Preferences, and Proportionality

Across the analysed documents, respect for autonomy and proportionality are recurrent ethical principles guiding the acceptable use of AI-supported recommendations.

International normative instruments emphasise that individuals should be informed about the use of AI and be able to exercise choices that reflect their goals of care [5,6]. Palliative interventions prioritise comfort, dignity, and individual goals rather than life-prolongation per se. Accordingly, AI outputs are described as adapted to patient preferences and tolerances and never deployed in a manner that introduces disproportionate burden relative to patient-centred goals of care [22,26].

This domain includes requirements for informed disclosure about AI involvement, mechanisms for obtaining consent (or surrogate decision-maker procedures where capacity is impaired), and routine reassessment of preferences as clinical trajectories evolve. These results are in line with prior ethical guidance proposed by the authors:

- Guideline 2: AI recommendations should be tailored to the patient’s preferences and limitations.
- Guideline 5: The informed consent of the patient and/or carer must be obtained.

(3) Transparency and Explainability

Transparency and explainability are described as foundational conditions for trust, accountability, and meaningful clinical deliberation. High-level frameworks (e.g., WHO and OECD) and technical–ethical scholarship argue that explainability enables clinicians to interrogate model outputs, justify decisions to patients, and support audit and regulatory review [5,7,18]. In palliative rehabilitation, where trade-offs between benefit and burden are subtle and personalised, inaccessible “black box” outputs can undermine clinical reasoning and patient trust.

These findings align with the ethical guidelines previously developed by the authors:

- Guideline 4: The choice of technology should be based on validated and transparent evidence.
- Guideline 7: The use of AI should be transparent and shared with the entire interdisciplinary team.

Building on this ethical foundation, the following transparency requirements were operationalised in the present analysis: (a) provision of clinically meaningful explanations tailored to clinician needs, (b) documentation of model versioning and data provenance, and (c) institutional procedures for recording how AI outputs informed each clinical decision.

Accordingly, transparency regarding AI involvement does not necessarily imply the need for separate consent beyond existing clinical consent procedures; rather, it requires meaningful disclosure proportionate to the role of AI in decision-making, particularly when AI outputs materially influence rehabilitation recommendations.

(4) Fairness, Equity, and Non-Discrimination

Justice-oriented concerns about biased datasets and unequal outcomes are repeatedly emphasised across international policy instruments and academic analyses [6,7].

AI models trained on non-representative data are identified as potentially producing recommendations that disadvantage older adults, people with disabilities, or socioeconomically marginalised patients—groups disproportionately represented in palliative settings [5].

For palliative rehabilitation, this domain requires proactive fairness assessments, monitoring for disparate impacts, and design/implementation choices that prioritise inclusive access (e.g., low-literacy interfaces, multilingual support).

Regulatory expectations increasingly include bias mitigation, outcome monitoring, and remedies when discriminatory impacts are detected.

This is consistent with the authors’ earlier published ethical guidelines:

- Guideline 6: The algorithms used must respect the principles of fairness and equity in access.

(5) Professional Competence and Ethical Literacy

The ethical deployment of AI depends on the competence and ethical awareness of the workforce and the institutions that support them. International guidance and implementation literature highlight the need for workforce development, organisational responsibility for training, and embedding ethical reflection into clinical practice [6,20].

In palliative care rehabilitation, clinicians must be literate not only in tool operation but also in algorithmic limitations, appropriate interpretation of outputs, and communication strategies that preserve humanisation of care [5,20].

The reviewed documents emphasise the need for continuous education programmes, competency standards for AI use, organisational responsibility for training, and multidisciplinary forums for ethical deliberation.

These findings are consistent with the authors’ previously published ethical guidelines:

- Guideline 8: The training of professionals in digital literacy and algorithmic ethics must be ongoing.

Although the domains were deductively informed by previously published ethical guidelines, their relevance and coherence were supported through coding of the analysed institutional documents. During this process, conceptual overlaps among several of the original guidelines were identified, leading to their consolidation into five broader ethical–regulatory domains that more closely reflect the structure of the governance principles identified in the analysed corpus.

Each domain maps onto multiple sections of the analysed corpus (policy statements, governance checklists, and ethical frameworks), providing robustness and traceability for the proposed ethical–regulatory norms. The domains also reflect a pragmatic orientation. They translate high-level ethical values (beneficence, non-maleficence, autonomy, justice) into regulatory-relevant requirements and clinical practices that are directly applicable to palliative rehabilitation.

A detailed mapping of the ethical and regulatory themes identified across the analysed institutional documents is presented in Supplementary Material S3.

### 3.2. Consolidated Ethical–Regulatory Guidelines for AI in Palliative Care Rehabilitation

Building on the ethical–regulatory domains identified through the documentary analysis, a set of consolidated ethical–regulatory guidelines was derived. These guidelines represent the analytical synthesis of ethical principles, governance requirements, and implementation-related provisions recurrently identified across the analysed institutional documents. The development of the consolidated guidelines involved aligning the ethical foundations identified in the documentary corpus with corresponding regulatory expectations described in international governance instruments. This alignment was then translated into practical requirements for clinical and organisational implementation in palliative care rehabilitation contexts.

Rather than introducing new ethical principles, the consolidated guidelines translate existing high-level ethical guidance into regulatory-relevant and clinically applicable requirements. This translation was achieved by mapping ethical values to corresponding regulatory implications and practical requirements, structured across the five ethical–regulatory domains identified in the analysis.

Table 1 presents the resulting consolidated ethical–regulatory guidelines. Each row reflects the analytical alignment between an ethical principle identified in the documentary corpus, the associated regulatory implication articulated in governance documents, and the corresponding practical requirement for clinical implementation.

The guidelines reflect convergent themes across international governance documents and function as a normative bridge between abstract ethical principles and operational regulatory considerations, supporting responsible, proportionate, and human-centred integration of AI technologies in palliative care rehabilitation settings.

The practical requirements presented in Table 1 constitute the consolidated ethical–regulatory guidance derived from the documentary analysis.

Together, the identified ethical–regulatory domains and the consolidated guidance presented in Table 1 provide the analytical basis for the interpretation and discussion of the implications of AI-supported rehabilitation in palliative care.

## 4. Discussion

Rather than reiterating the descriptive presentation of the ethical–regulatory domains provided in Section 3, the following discussion focuses on interpreting their implications for AI governance, clinical accountability, and decision-making in palliative care rehabilitation.

The present study synthesised previously published ethical guidance into ethical–regulatory guidelines applicable to the use of artificial intelligence (AI) in palliative care rehabilitation. Through qualitative documentary analysis and thematic synthesis of international governance and policy documents, five ethical–regulatory domains were identified and translated into practical guidance that reflects both ethical principles and regulatory expectations. The resulting framework responds to a recognised gap between high-level AI ethics and the operational realities of ethically sensitive clinical contexts such as palliative rehabilitation.

Unlike many existing approaches that rely primarily on non-binding ethical principles or broad regulatory statements, the present analysis contributes to ongoing discussions by articulating how ethical concerns can be operationalised in a regulatory-relevant and clinically meaningful manner. By grounding the analysis in authoritative international documents and aligning it with previously published ethical guidelines, the study offers a structured and context-sensitive contribution that complements, rather than duplicates, existing AI governance efforts.

### 4.1. Interpretation of the Ethical–Regulatory Domains

Human Oversight and Clinical Responsibility

The centrality of human oversight and clinical responsibility identified in this analysis is consistent with a broad body of international AI ethics and governance literature, which frames AI in healthcare primarily as a decision-support technology rather than a substitute for professional judgement [5,8,18].

Concepts such as human-in-the-loop and human-on-the-loop supervision are frequently referenced in international governance discussions and reflect a shared concern with accountability, traceability, and the attribution of responsibility for clinical outcomes. Within contemporary AI governance, human-in-the-loop supervision refers to arrangements in which a qualified human professional remains actively involved in the decision-making process, with the capacity to review, modify, or override algorithmic outputs before they are acted upon. In contrast, human-on-the-loop supervision describes oversight mechanisms whereby a human monitors AI system performance and outcomes, retaining the authority to intervene when predefined thresholds, risks, or anomalies are identified, even if decisions are executed automatically in routine cases. Both models are recognised in European and international regulatory discussions as mechanisms to ensure accountability, traceability, and the preservation of human responsibility in high-risk domains such as healthcare [5,7,26].

While these principles are widely acknowledged at a general level, existing guidance rarely addresses how human oversight should be operationalised in clinically complex and ethically sensitive settings such as palliative care rehabilitation. In this context, clinical decision-making is characterised by uncertainty, rapid changes in patient status, and value-laden trade-offs that cannot be reduced to algorithmic optimisation.

The ethical–regulatory guidance consolidated in this study contributes to existing frameworks by highlighting the need for concrete organisational mechanisms—such as explicit role definition, documentation of AI-supported decisions, and escalation pathways when clinical judgement diverges from algorithmic outputs—to ensure that human responsibility is preserved in practice, not merely in principle.

For example, in a palliative rehabilitation setting where an AI system recommends increasing exercise intensity based on functional performance metrics, the clinician must retain the authority to contextualise this recommendation in light of the patient’s symptom burden, fatigue levels, and personal goals of care.

Patient Autonomy, Preferences, and Proportionality

Respect for patient autonomy and proportionality has long been recognised as a cornerstone of both biomedical ethics and palliative care practice [10,27]. International AI governance frameworks similarly emphasise informed consent, alignment with intended medical purpose, and proportionality between system risk and safeguards [6,17]. The findings of this study reinforce the relevance of these principles while illustrating their heightened significance in palliative rehabilitation contexts.

Unlike many areas of acute or preventive medicine, palliative care prioritises comfort, dignity, and individual goals over disease modification. Despite this, the literature on AI ethics offers limited discussion of how proportionality should be interpreted when benefits are subjective, burdens are cumulative, and patient preferences may evolve over time. This perspective aligns with palliative care literature emphasising that the perceived burden of an intervention is subjective, context-dependent, and may change over time, underscoring the need for continuous ethical reassessment rather than one-time consent [10,11].

The ethical–regulatory guidance derived from this domain seeks to link AI-supported rehabilitation decisions to ongoing assessment of patient goals, functional tolerance, and perceived burden, rather than static notions of clinical effectiveness alone.

In this sense, AI systems introduce an additional layer of mediation between clinicians and patients, requiring that transparency about the role of algorithmic recommendations becomes part of the broader process of informed decision-making.

For instance, an AI-generated rehabilitation plan suggesting daily mobility exercises may appear clinically appropriate based on functional data yet remain ethically inappropriate if the patient prioritises comfort and minimal physical burden during advanced stages of illness.

Transparency and Explainability

Transparency and explainability are frequently cited as foundational requirements for trustworthy AI in healthcare, particularly to support accountability and regulatory oversight [7,18]. Empirical studies have also shown that lack of explainability can undermine clinician trust and hinder adoption of AI systems in clinical practice [28].

In palliative care rehabilitation, the need for explainability extends beyond regulatory compliance. Clinicians must be able to interpret and contextualise AI outputs in conversations with patients and families, where decisions are closely tied to personal values and expectations.

The findings of this study suggest that transparency should be understood primarily in terms of clinical meaningfulness rather than technical disclosure, reinforcing calls in the literature to tailor explainability to the needs of end-users rather than developers or regulators alone. Notably, few existing frameworks explicitly address this distinction in palliative or rehabilitative contexts, highlighting an area where further empirical research is needed.

This tension illustrates a broader challenge in the governance of clinical AI. Systems may satisfy formal transparency requirements while still failing to produce explanations that are meaningful for clinicians and patients in ethically sensitive care contexts.

In practice, this may involve situations where a clinician must explain to a patient or family that a rehabilitation recommendation was partly informed by an AI-supported decision-support system, clarifying how the recommendation was interpreted and adapted to the patient’s individual circumstances.

Fairness, Equity, and Non-Discrimination

Concerns regarding bias, data representativeness, and unequal outcomes are well documented in AI ethics and health policy literature [7,29,30]. These concerns are particularly relevant for palliative care populations, who are often older, multimorbid, and under-represented in datasets used to train AI systems, with overlapping and intersecting vulnerabilities that may compound the risk of bias and unequal outcomes.

While international policy documents consistently warn against the risk of exacerbating health inequalities, there is limited guidance on how fairness assessments should be implemented in rehabilitation or palliative care settings. The ethical–regulatory guidance consolidated in this study therefore contributes by foregrounding the need for proactive bias monitoring and inclusive design strategies tailored to vulnerable populations, rather than assuming that generic fairness measures are sufficient across all healthcare domains.

These concerns highlight the importance of continuous monitoring of AI-supported clinical outcomes to detect potential disparities in recommendations or effects across different patient groups, particularly in vulnerable populations commonly represented in palliative care settings.

For example, if an AI system used for rehabilitation planning is trained primarily on data from younger or non-palliative populations, its recommendations may inadvertently underestimate fatigue levels or functional limitations commonly experienced in advanced stages of illness.

Professional Competence and Ethical Literacy

The importance of professional competence and ethical literacy in AI deployment is increasingly recognised, with several frameworks highlighting organisational responsibility for training and governance [5,20]. However, much of this literature remains focused on technical literacy or general ethical awareness.

In palliative care rehabilitation, ethical competence encompasses not only understanding AI limitations but also the ability to integrate algorithmic recommendations into relational, person-centred care. The findings of this study align with calls to embed ethical reflection into routine clinical practice and extend them by emphasising the need for multidisciplinary dialogue and institutional support structures that recognise the emotional and moral complexity of palliative rehabilitation work.

In clinical practice, this may involve situations where professionals must critically assess whether an AI-generated exercise progression remains clinically and ethically appropriate as a patient’s condition deteriorates, requiring both technical understanding of the tool and ethical judgement regarding the proportionality of care.

These illustrative scenarios highlight how abstract ethical principles identified in international governance frameworks may translate into concrete ethical considerations within everyday clinical decision-making in palliative care rehabilitation.

### 4.2. Implications for Clinical Practice and Governance

The ethical–regulatory guidance consolidated in this study has several implications for clinical practice, institutional governance, and regulatory oversight of AI-supported rehabilitation in palliative care. Rather than prescribing specific technologies or clinical protocols, the guidance provides a structured reference to support decision-making in contexts characterised by heightened vulnerability, uncertainty, and ethical complexity.

From a clinical perspective, the findings highlight the importance of embedding AI systems within existing professional responsibilities and care pathways. In palliative care rehabilitation, these ethical–regulatory considerations are especially salient when AI tools are used to inform exercise intensity, progression, or discontinuation decisions. The emphasis on human oversight, proportionality, and explainability suggests that AI-supported rehabilitation tools should be integrated as adjuncts to clinical reasoning, not as substitutes for professional judgement. In practice, this implies that clinicians require not only access to AI-generated recommendations but also the institutional support needed to interpret, contextualise, and, when appropriate, override algorithmic outputs. Such support is particularly relevant in palliative care rehabilitation, where clinical decisions are closely intertwined with patient values, symptom burden, and evolving goals of care.

At the organisational level, the guidance underscores the role of healthcare institutions in operationalising ethical and regulatory expectations. This includes establishing clear governance structures for AI use, defining roles and responsibilities, and ensuring documentation and traceability of AI-supported decisions. Training and ethical literacy emerge as critical enablers of responsible implementation, reinforcing calls in the literature for continuous professional development that goes beyond technical competence to include ethical reflection and communication skills. In palliative care settings, where interdisciplinary collaboration is central, governance mechanisms should also facilitate dialogue across professional groups to address ethical tensions arising from AI-supported decision-making.

The findings also have implications for policymakers and regulators. As regulatory frameworks increasingly adopt risk-based approaches to AI governance, the present analysis illustrates how high-level principles may be translated into context-sensitive expectations for ethically complex domains such as palliative care rehabilitation. The ethical–regulatory domains identified in this study align with existing international governance frameworks while providing additional specificity regarding vulnerable populations and relational care contexts. This specificity may inform the development of sectoral guidance, standards, or implementation tools that complement overarching regulatory instruments without imposing rigid or inappropriate requirements.

From an organisational perspective, the responsible implementation of AI-supported rehabilitation tools also requires the integration of these systems within existing governance and accountability structures. Healthcare institutions may need to establish clear internal procedures specifying how AI-assisted recommendations are validated, documented, and reviewed within clinical workflows. Such procedures can help ensure that algorithm-informed decisions remain traceable and aligned with professional responsibilities. Embedding AI use within institutional governance frameworks may also facilitate multidisciplinary oversight and support ongoing evaluation of how AI-supported recommendations influence clinical decision-making in this context.

Finally, the study highlights the importance of recognising palliative care rehabilitation as a distinct clinical context within broader AI governance discussions. Many existing frameworks adopt a generalised view of healthcare and may not explicitly address the ethical nuances associated with end-of-life care, fluctuating capacity, and the prioritisation of dignity and comfort. By articulating ethical–regulatory implications tailored to this context, the present guidance contributes to more nuanced and responsive approaches to AI governance in healthcare.

### 4.3. Limitations and Future Directions

This study has some limitations that should be acknowledged when interpreting its findings.

First, the analysis was based on a focused corpus of nine institutional documents issued by major international and supranational organisations. While this selection reflects an intentional emphasis on highly authoritative and widely recognised governance sources, it may limit the breadth of perspectives included in the analysis. In particular, regional- or institutional-level regulatory frameworks were not examined and may offer additional contextual insights across different healthcare systems.

Consequently, the ethical–regulatory guidance proposed in this study should be interpreted as a conceptual and governance-oriented framework rather than as a universally applicable regulatory model across all healthcare systems.

Second, although academic literature was consulted to support conceptual contextualisation, the study did not aim to conduct an exhaustive systematic review of empirical evidence on the use of artificial intelligence in rehabilitation or palliative care. The role of academic sources was deliberately limited to contextual validation rather than domain generation, reflecting the normative and regulatory focus of the analysis. Consequently, empirical outcomes related to the effectiveness, safety, or acceptability of specific AI-supported rehabilitation interventions were not examined.

Third, the guidance was developed through documentary analysis and normative synthesis rather than stakeholder engagement. Perspectives from patients, caregivers, clinicians, developers, and regulators were not directly incorporated into the analytical process. While the guidance is grounded in internationally endorsed frameworks, future work would benefit from participatory approaches that explore how ethical–regulatory principles are understood, prioritised, and negotiated in real-world palliative care rehabilitation settings.

Future research could build on these findings in several ways. Empirical studies examining the implementation of AI-supported rehabilitation tools in palliative care contexts could assess how ethical–regulatory considerations influence clinical decision-making, patient experience, and professional practice. Qualitative research involving clinicians, patients, and caregivers may further illuminate how concepts such as proportionality, autonomy, and explainability are interpreted in practice and how AI systems interact with relational aspects of care.

In addition, comparative analyses across jurisdictions could explore how evolving regulatory frameworks—such as risk-based approaches to AI governance—are operationalised in ethically sensitive healthcare domains. Finally, as AI technologies evolve, regulatory instruments will also continue to develop, making longitudinal research important to assess how ethical–regulatory guidance adapts over time and how emerging challenges are addressed in palliative care rehabilitation.

## 5. Conclusions

This study presents context-sensitive ethical–regulatory guidance for the use of artificial intelligence (AI) in palliative care rehabilitation by translating high-level international ethical and governance principles into applied clinically relevant domains.

Through a structured documentary analysis and thematic synthesis, five ethical–regulatory domains were identified—human oversight and clinical responsibility; patient autonomy, preferences, and proportionality; transparency and explainability; fairness, equity, and non-discrimination; and professional competence and ethical literacy. Together, these domains provide a structured framework that bridges the gap between abstract AI ethics and the operational realities of ethically sensitive rehabilitation practice.

By grounding the analysis in authoritative international policy and regulatory documents and aligning it with previously published ethical guidance, this work contributes to ongoing discussions on pragmatic approaches to AI governance that are responsive to the complexity, vulnerability, and relational dimensions of palliative care. Rather than proposing new ethical principles, the study demonstrates how existing norms can be systematised and operationalised to support responsible clinical decision-making and institutional accountability in high-risk contexts.

The proposed ethical–regulatory guidance is intended to support clinicians, healthcare organisations, and policymakers in navigating the ethical and regulatory challenges associated with AI-supported rehabilitation in palliative care. By emphasising proportionality, human responsibility, and context-sensitive implementation, the framework seeks to safeguard patient dignity and autonomy while enabling the careful and appropriate integration of AI technologies into clinical practice.

By translating high-level AI governance principles into operational ethical–regulatory guidance, this study makes it possible to more systematically integrate AI-supported rehabilitation tools into clinically accountable and ethically grounded care processes in palliative settings.

As AI technologies continue to evolve and regulatory frameworks further mature, context-specific ethical–regulatory approaches such as the one presented here will be increasingly important. Future research should focus on empirical validation, stakeholder engagement, and adaptation of the guidance across jurisdictions to ensure that AI deployment in palliative care rehabilitation remains ethically grounded, clinically meaningful, and responsive to the needs of vulnerable patient populations.

## Figures and Tables

**Table 1:** Consolidated ethical–regulatory guidelines for the use of artificial intelligence in palliative care rehabilitation. The table summarises the ethical–regulatory domains identified through documentary analysis and presents the corresponding ethical basis, associated regulatory implications, and practical requirements for clinical implementation. The consolidated guidelines reflect the analytical synthesis of international governance and policy documents and translate high-level ethical principles into regulatory-relevant and context-sensitive guidance for palliative care rehabilitation.

Ethical–Regulatory Domain	Ethical Basis	Regulatory Implication	Practical Requirement for Clinical Implementation
Human Oversight and Clinical Responsibility	Grounded in principles of beneficence, non-maleficence, accountability, and professional responsibility, emphasising the moral and clinical responsibility to retain human judgement in care delivery.	AI systems used in healthcare are associated with requirements for human-in-the-loop or human-on-the-loop oversight, clear attribution of responsibility, and traceability of AI-supported decisions throughout the clinical workflow.	AI-supported rehabilitation recommendations should be reviewed and validated by qualified healthcare professionals; roles and responsibilities for AI use should be clearly defined; AI outputs and clinician decisions should be documented in the clinical record.
Patient Autonomy, Preferences, and Proportionality	Anchored in respect for autonomy, dignity, informed consent, and proportionality, particularly relevant in contexts of vulnerability and fluctuating decision-making capacity.	Regulatory frameworks emphasise alignment between AI system use, intended medical purpose, and patient goals of care, with proportional safeguards based on risk and context of use.	Patients and/or caregivers should be informed about the involvement of AI in rehabilitation decisions; AI-supported interventions should be adapted to patient preferences, functional status, and goals of care; proportionality between expected benefit and burden should be regularly reassessed.
Transparency and Explainability	Based on principles of transparency, explicability, accountability, and trust, enabling meaningful clinical deliberation and patient engagement.	AI systems are subject to documentation, traceability, and transparency requirements across the system lifecycle, supporting auditability and regulatory oversight.	Clinicians should have access to understandable and clinically meaningful explanations of AI outputs; documentation of data sources, model purpose, and updates should be available; AI involvement in clinical decisions should be transparent to patients and care teams.
Fairness, Equity, and Non-Discrimination	Rooted in principles of justice, equity, and non-discrimination, with particular attention to vulnerable and under-represented populations.	Regulatory guidance highlights the need for bias assessment, data quality assurance, and monitoring of potential disparate impacts associated with AI use in healthcare.	AI-supported rehabilitation systems should be evaluated for potential bias affecting palliative care populations; inclusive design and deployment strategies should be considered and prioritised; outcomes should be monitored to detect and address inequitable effects.
Professional Competence and Ethical Literacy	Informed by principles of professional integrity, responsibility, and ethical competence, recognising the responsibilities of clinicians and institutions in responsible AI use.	Governance frameworks emphasise organisational responsibility for training, competency development, and ethical awareness among AI users.	Healthcare organisations should provide or support ongoing training in AI literacy and ethics; clinicians should be supported in understanding AI limitations and appropriate use; multidisciplinary forums for ethical reflection and governance should be encouraged.

Note: The guidelines presented reflect an analytical synthesis of existing international ethical and regulatory frameworks and are intended to support clinical governance and decision-making. They do not constitute legally binding requirements.

## Data Availability

The original contributions presented in this study are included in the article and Supplementary Materials. Further inquiries can be directed to the corresponding author.

## Supplementary Materials

The following supporting information can be downloaded at https://www.mdpi.com/article/10.3390/healthcare14070895/s1, Supplementary Materials S1: list of the institutional documents included in the analysis; Supplementary Materials S2: detailed search strategies used for academic database consultation (PubMed/MEDLINE, Scopus, and Web of Science); Supplementary Materials S3: structured data extraction database table.

## Abbreviations

The following abbreviations are used in this manuscript:

AI	Artificial Intelligence
EU	European Union
OECD	Organisation for Economic Co-operation and Development
WHO	World Health Organization
