# Supplementary material for: Ethical–Regulatory Guidelines for AI in Palliative Care Rehabilitation
Source: Healthcare (Basel). 2026 Mar 31;14(7):895. doi: 10.3390/healthcare14070895 (PMC13072931; doi:10.3390/healthcare14070895)
Supplement: Supplementary file 1 [file healthcare-14-00895-s001.zip › Supplementary Material S3.pdf]

Ethical regulatory guidelines for AI in palliative care rehabilitation

Daniela Oliveira, Sofia Nunes , Francisca Rego and Rui Nunes

Supplementary Material S3

Title	year of publication	author	type	Geographical scope	keywords	Ethical-regulatory domain	Regulatory or governance elements	ethical principles	extracted idea/citations	Regulatory implication	Implications for palliative care rehabilitation	Link to guideline (Yes / Which)	Notes	Link reference
Recommendation on the Ethics of AI	2021	Unesco	International ethical guideline	Global	AI; ethics; guidelines;	Human Oversight and Clinical Responsibility	professional responsability; oversight requirements	Responsibility; Beneficence; Non-maleficence	AI systems should be designed and used under appropriate human oversight to ensure accountability and responsibility for outcomes.	Requirement for human-in-the-loop or human-on-the-loop mechanisms and clear attribution of responsibility for AI-supported decisions	Ensures that AI-supported rehabilitation or exercise recommendations are always supervised and validated by healthcare professionals in vulnerable palliative care contexts.	guideline 1 and 3 - AI should complement—and never replace—human clinical judgement.. There should be constant clinical supervision of the exercises suggested by AI	Section on Human Oversight and Responsibility (approx. pp. 23–24)	https://unesdoc.unesco.org/ark:/48223/pf0000381137
Recommendation on the Ethics of Artificial Intelligence	2021	Unesco	International ethical guideline	Global	AI; ethics; guidelines;	Patient Autonomy, Preferences, and Proportionality	professional responsability; accountability mechanisms	Autonomy & Consent; Dignity; Fairness & non-discrimination	The dignity, autonomy, and rights of individuals must be respected throughout the AI system lifecycle, particularly for vulnerable populations.	Obligation to ensure informed consent, respect for personal values, and safeguards for individuals with reduced decision-making capacity. Risk-based regulatory approach; pre-specification of intended medical purpose; proportionality between AI system risk and regulatory requirements; consideration of context and population in which AI is deployed.	Supports person-centred rehabilitation decisions aligned with patient goals of care and limits the use of AI-driven interventions that may be disproportionate. Definition and documentation of intended use; limitation of AI deployment to contexts where benefits outweigh potential burdens; ongoing reassessment of appropriateness of AI-supported interventions, particularly in vulnerable or high-risk populations.	guideline 2 and 5- AI recommendations should be tailored to the patient's preferences and limitations. The informed consent of the patient and/or carer must be obtained.	Human Rights and Dignity section (approx. pp. 14–16)	https://unesdoc.unesco.org/ark:/48223/pf0000381137
Recommendation on the Ethics of Artificial Intelligence	2021	Unesco	International ethical guideline	Global	AI; ethics; guidelines;	Transparency and Explainability	auditability; risk classification	Transparency & explainability; Safety & robustness; Accountability	AI systems should be transparent and explainable, enabling understanding of their functioning and outcomes by relevant stakeholders.	Expectation that AI systems used in healthcare provide understandable explanations and support auditability and traceability. Mandatory documentation across the total AI lifecycle; traceability of development decisions; transparency regarding data sources, model updates, and validation processes; documentation proportional to system risk.	Allows clinicians to understand and explain AI-supported rehabilitation recommendations to patients and caregivers, fostering trust and informed decision-making. Clear documentation of datasets, model design, validation, and updates; availability of information to regulators and end-users; traceable records enabling audit, regulatory review, and clinical accountability.	guideline 4 and 7- The choice of technology should be based on validated and transparent evidence; The use of AI should be transparent and shared with the entire interdisciplinary team.	Transparency and Explainability section (approx. pp. 18–19)	https://unesdoc.unesco.org/ark:/48223/pf0000381137
Recommendation on the Ethics of Artificial Intelligence	2021	Unesco	International ethical guideline	Global	AI; ethics; guidelines;	Fairness, Equity, and Non-Discrimination	oversight requirements; acountability mechanisms	Justice; Equity; Non-discrimination	AI systems should not exacerbate inequalities and should promote fairness and inclusion, particularly for marginalised or vulnerable groups.	Need for bias assessment, monitoring of discriminatory outcomes, and inclusive design and deployment of AI systems.	Helps prevent unequal access to AI-supported rehabilitation services among patients with disabilities, cognitive impairment, or limited digital access.	guideline 6-The algorithms used must respect the principles of fairness and equity in access.	Fairness and Non-Discrimination section (approx. pp. 20–21)	https://unesdoc.unesco.org/ark:/48223/pf0000381137
Recommendation on the Ethics of Artificial Intelligence	2021	Unesco	International ethical guideline	Global	AI; ethics; guidelines;	Professional Competence and Ethical Literacy	professional responsability	Professional responsibility; transparency & explainability	States and organisations should promote education, training, and ethical awareness regarding AI use among professionals.	Institutional responsibility to ensure adequate training and ethical competence for professionals using AI in healthcare.	Supports the need for trained rehabilitation professionals capable of critically and ethically using AI tools in sensitive palliative care settings.	guideline 8- The training of professionals in digital literacy and algorithmic ethics must be ongoing.	Education and Capacity Building section (approx. pp. 26–27)	https://unesdoc.unesco.org/ark:/48223/pf0000381137
Artificial intelligence in healthcare - Applications, risks, and ethical and societal impacts	2022	European Parliamentary Research Service (EPRS)	Policy and regulatory analysis report	EU	AI; EU; risks; ethics; impact	Human Oversight and Clinical Responsability	Oversight requirements;	dignity; acountability	The report discusses AI primarily as a tool to support clinical decision-making in healthcare and highlights ongoing concerns regarding professional responsibility and accountability when AI systems are used. "Furthermore, future AI solutions in healthcare must be dynamic, i.e., they should be embedded with mechanisms to continue to learn from new scenarios and mistakes as they are detected in practice. However, this last aspect will still require a certain degree of human control and vigilance to identify problems as they appear;"	Requirement for human-in-the-loop or human-on-the-loop mechanisms and clear attribution of responsibility for AI-supported decisions	Ensures that AI-supported rehabilitation or exercise recommendations are always supervised and validated by healthcare professionals in vulnerable palliative care contexts.	guideline 1 and 3 - AI should complement—and never replace—human clinical judgement.. There should be constant clinical supervision of the exercises suggested by AI	Discussion on clinical decision support systems and liability in healthcare contexts. (pp.17)	Artificial intelligence in healthcare: Applications, risks, and ethical and societal impacts | Think Tank | European Parliament
Artificial intelligence in healthcare Applications, risks, and ethical and societal impacts	2022	European Parliamentary Research Service (EPRS)	Policy and regulatory analysis report	EU	AI; EU; risks; ethics; impact	Fairness, Equity, and Non-Discrimination	oversight requirements; acountability mechanisms	Justice; Equity; Non-discrimination	Despite continuous advances in medical research and healthcare delivery, there remain important inequalities and inequities in medical care within most countries around the world. The main factors that contribute to these inequalities and inequities include sex/gender, age, ethnicity, income, education and geography.	Need for bias assessment, monitoring of discriminatory outcomes, and inclusive design and deployment of AI systems.	Helps prevent unequal access to AI-supported rehabilitation services among patients with disabilities, cognitive impairment, or limited digital access.	guideline 6-The algorithms used must respect the principles of fairness and equity in access.	Risk of bias in medical AI and perpetuation of inequities (pp. 20)	Artificial intelligence in healthcare: Applications, risks, and ethical and societal impacts | Think Tank | European Parliament
Artificial intelligence in healthcare Applications, risks, and ethical and societal impacts	2022	European Parliamentary Research Service (EPRS)	Policy and regulatory analysis report	EU	AI; EU; risks; ethics; impact	Transparency and Explainability	auditability; risk classification	Transparency & explainability; Safety & robustness; Accountability	The report highlights transparency as a key challenge in AI-enabled healthcare, noting the importance of patients and healthcare professionals being aware of the involvement of AI systems in clinical decision-making processes. " In practice, existing AI tools in healthcare are rarely delivered with full traceability. In fact, companies often prefer not to disclose too much information about their algorithms, which are thus delivered as opaque tools that are difficult to understand and examine by independent parties. This, in turn, reduces the level of trust and adoption into real-world practice."	Expectation that AI systems used in healthcare provide understandable explanations and support auditability and traceability. Mandatory documentation across the total AI lifecycle; traceability of development decisions; transparency regarding data sources, model updates, and validation processes; documentation proportional to system risk.	Allows clinicians to understand and explain AI-supported rehabilitation recommendations to patients and caregivers, fostering trust and informed decision-making. Clear documentation of datasets, model design, validation, and updates; availability of information to regulators and end-users; traceable records enabling audit, regulatory review, and clinical accountability.	guideline 4 and 7- The choice of technology should be based on validated and transparent evidence; The use of AI should be transparent and shared with the entire interdisciplinary team.	Lack of transparency (pp.21 - 23)	Artificial intelligence in healthcare: Applications, risks, and ethical and societal impacts | Think Tank | European Parliament

Artificial intelligence in healthcare Applications, risks, and ethical and societal impacts	2022	European Parliamentary Research Service (EPRS)	Policy and regulatory analysis report	EU	AI; EU; risks; ethics; impact	Patient Autonomy, Preferences, and Proportionality	professional responsability; accountability mechanisms	Autonomy & Consent; Dignity; Fairness & non-discrimination	The report enfasises the increased difficulty to maintain the information clear and explainable so the patients and caregivers are confident enough to give their consent: "It has become increasingly difficult for patients to understand the decision-making process and the different ways in which their data can be reused, and to know exactly how they can choose to opt out of sharing their data. Issues of informed consent are also especially prominent in big data research, especially digital platform-based health data research, in which a patient may not be fully aware of or fully understand the extent to which their data is shared and reused"	Obligation to ensure informed consent, respect for personal values, and safeguards for individuals with reduced decision-making capacity. Risk-based regulatory approach; pre-specification of intended medical purpose; proportionality between AI system risk and regulatory requirements; consideration of context and population in which AI is deployed.	Supports person-centred rehabilitation decisions aligned with patient goals of care and limits the use of AI-driven interventions that may be disproportionate. Definition and documentation of intended use; limitation of AI deployment to contexts where benefits outweigh potential burdens; ongoing reassessment of appropriateness of AI-supported interventions, particularly in vulnerable or high-risk populations.	guideline 2 and 5- AI recommendations should be tailored to the patient's preferences and limitations. The informed consent of the patient and/or carer must be obtained.	3.5. Privacy and security issues (pp.23-25)	Artificial intelligence in healthcare: Applications, risks, and ethical and societal impacts | Think Tank | European Parliament
Artificial intelligence in healthcare Applications, risks, and ethical and societal impacts	2022	European Parliamentary Research Service (EPRS)	Policy and regulatory analysis report	EU	AI; EU; risks; ethics; impact	Professional Competence and Ethical Literacy	professional responsability	Professional responsibility; transparency & explainability	The report identifies professional training, organisational preparedness, and governance structures as key challenges for the effective and ethical implementation of AI systems in healthcare. " it is not clear that medical AI tools will be systematically interoperable across clinical sites and health systems, and that they will be easily integrated within existing clinical and technical workflows (Meskó & Görög, 2020), without significant modifications to existing clinical practices, care models and even training programmes."	Institutional responsibility to ensure adequate training and ethical competence for professionals using AI in healthcare.	Supports the need for trained rehabilitation professionals capable of critically and ethically using AI tools in sensitive palliative care settings.	guideline 8- The training of professionals in digital literacy and algorithmic ethics must be ongoing.	Grounds ethical literacy requirements in real-world implementation challenges.. (3.7. Obstacles to implementation in real-world healthcare (pp. 28-29))	Artificial intelligence in healthcare: Applications, risks, and ethical and societal impacts | Think Tank | European Parliament
The Impact of Artificial Intelligence on Health Outcomes for Key Populations: Navigating Health Inequalities in the EU – Final Joint Statement	2023	EU Health Policy Platform – Thematic Network on AI and Health Inequalities (coordinated by Brunel University London Centre for Artificial Intelligence and Health Action International; endorsed by multiple academic, legal, and civil society organisations)	Joint policy statement / ethical–regulatory position paper	EU	AI; healthcare; health inequalities; vulnerable populations; fundamental rights	Fairness, Equity, and Non-Discrimination	oversight requirements; accountability mechanisms	Fairness & non-discrimination; justice; protection of vulnerable populations	Health data are often [...] non-representative and non-generalisable (e.g. because of lack of inclusive clinical data, underrepresentation of minorities). " Promote inclusion by design as well as ethical principles among public as well as private AI developing organisations in alignment with a regulatory framework. "	Institutional and regulatory obligation to prevent algorithmic bias and ensure that AI systems do not exacerbate existing health inequalities through biased or incomplete datasets.	Highly relevant to palliative care rehabilitation, where patients are frequently underrepresented in datasets and at increased risk of exclusion from data-driven decision-making.	guideline 6-The algorithms used must respect the principles of fairness and equity in access.	pp. 3 and pp11	Joint Statement on the Impact of Artificial Intelligence on Health Outcomes for Key Populations: Navigating Health Inequalities in the EU - Health Action International
The Impact of Artificial Intelligence on Health Outcomes for Key Populations: Navigating Health Inequalities in the EU – Final Joint Statement	2023	EU Health Policy Platform – Thematic Network on AI and Health Inequalities (coordinated by Brunel University London Centre for Artificial Intelligence and Health Action International; endorsed by multiple academic, legal, and civil society organisations)	Joint policy statement / ethical–regulatory position paper	EU	AI; healthcare; health inequalities; vulnerable populations; fundamental rights	Human Oversight and Clinical Responsibility	institutional and regulatory responsibility; Accountability; human oversight;	Responsibility; Beneficence; Non-maleficence	"There is a lack of guidelines and scientific standards to use health data as training data."; "AI used in healthcare deserves special consideration because personal and public health is at stake and individuals are in a vulnerable position when in need of healthcare".	Requirement for human-in-the-loop or human-on-the-loop mechanisms and clear attribution of responsibility for AI-supported decisions	Ensures that AI-supported rehabilitation or exercise recommendations are always supervised and validated by healthcare professionals in vulnerable palliative care contexts.	guideline 1 and 3 - AI should complement—and never replace—human clinical judgement.. There should be constant clinical supervision of the exercises suggested by AI.	pp. 3; pp. 9	Joint Statement on the Impact of Artificial Intelligence on Health Outcomes for Key Populations: Navigating Health Inequalities in the EU - Health Action International
The Impact of Artificial Intelligence on Health Outcomes for Key Populations: Navigating Health Inequalities in the EU – Final Joint Statement	2023	EU Health Policy Platform – Thematic Network on AI and Health Inequalities (coordinated by Brunel University London Centre for Artificial Intelligence and Health Action International; endorsed by multiple academic, legal, and civil society organisations)	Joint policy statement / ethical–regulatory position paper	EU	AI; healthcare; health inequalities; vulnerable populations; fundamental rights	Professional Competence and Ethical Literacy	institutional and professional responsibility	Professional responsibility; transparency & explainability	"Health data are often [...] unstructured, incomplete [...] and subject to measurement errors."; "Acknowledging the need for special attention for the effects of AI used in healthcare for human health, wellbeing and fundamental rights". " Involve people with lived experience in all stages of the development of AI, using a community centred approach and guaranteeing representation of key populations not only in datasets but also in developer teams."	Institutional responsibility to ensure adequate training and ethical competence for professionals using AI in healthcare.	Supports the need for trained rehabilitation professionals capable of critically and ethically using AI tools in sensitive palliative care settings.	guideline 8- The training of professionals in digital literacy and algorithmic ethics must be ongoing.	pp. 3 and pp. 9	Joint Statement on the Impact of Artificial Intelligence on Health Outcomes for Key Populations: Navigating Health Inequalities in the EU - Health Action International

The Impact of Artificial Intelligence on Health Outcomes for Key Populations: Navigating Health Inequalities in the EU – Final Joint Statement	2023	EU Health Policy Platform – Thematic Network on AI and Health Inequalities (coordinated by Brunel University London Centre for Artificial Intelligence and Health Action International; endorsed by multiple academic, legal, and civil society organisations)	Joint policy statement / ethical–regulatory position paper	EU	AI; healthcare; health inequalities; vulnerable populations; fundamental rights	Transparency and Explainability	auditability; risk classification	Transparency & explainability; Safety & robustness; Accountability	" 10.Introducing a separate category for medical devices using AI in the EUDAMED database to create more awareness about the use of AI in medical devices and improve transparency. 11. Require all health AI systems used in the EU, not merely high-risk systems, to be registered in the EU public database which will be developed under the European AI Act to increase transparency and include information on the users of the system (institutions which use a particular system e.g., a specific hospital) and any impact assessments carried out.	Expectation that AI systems used in healthcare provide understandable explanations and support auditability and traceability. Mandatory documentation across the total AI lifecycle; traceability of development decisions; transparency regarding data sources, model updates, and validation processes; documentation proportional to system risk.	Allows clinicians to understand and explain AI-supported rehabilitation recommendations to patients and caregivers, fostering trust and informed decision-making. Clear documentation of datasets, model design, validation, and updates; availability of information to regulators and end-users; traceable records enabling audit, regulatory review, and clinical accountability.	guideline 4 and 7- The choice of technology should be based on validated and transparent evidence; The use of AI should be transparent and shared with the entire interdisciplinary team.		Joint Statement on the Impact of Artificial Intelligence on Health Outcomes for Key Populations: Navigating Health Inequalities in the EU - Health Action International
The Impact of Artificial Intelligence on Health Outcomes for Key Populations: Navigating Health Inequalities in the EU – Final Joint Statement	2023	EU Health Policy Platform – Thematic Network on AI and Health Inequalities (coordinated by Brunel University London Centre for Artificial Intelligence and Health Action International; endorsed by multiple academic, legal, and civil society organisations)	Joint policy statement / ethical–regulatory position paper	EU	AI; healthcare; health inequalities; vulnerable populations; fundamental rights	Patient Autonomy, Preferences, and Proportionality	professional responsability; accountability mechanisms	Autonomy & Consent; Dignity; Fairness & non-discrimination	It is critical to develop regulatory mechanisms that foster patient need-driven innovation [...] for populations who have historically been overlooked.". " "Ensuring robust protection of personal data and confidentiality to ensure that personal data obtained in the context of receiving care is not further shared or in particular, for secondary uses outside the provision of healthcare. "	Obligation to ensure informed consent, respect for personal values, and safeguards for individuals with reduced decision-making capacity. Risk-based regulatory approach; pre-specification of intended medical purpose; proportionality between AI system risk and regulatory requirements; consideration of context and population in which AI is deployed.	Supports person-centred rehabilitation decisions aligned with patient goals of care and limits the use of AI-driven interventions that may be disproportionate. Definition and documentation of intended use; limitation of AI deployment to contexts where benefits outweigh potential burdens; ongoing reassessment of appropriateness of AI-supported interventions, particularly in vulnerable or high-risk populations.	guideline 2 and 5- AI recommendations should be tailored to the patient's preferences and limitations. The informed consent of the patient and/or carer must be obtained.	pp.3 and pp.11	Joint Statement on the Impact of Artificial Intelligence on Health Outcomes for Key Populations: Navigating Health Inequalities in the EU - Health Action International
Regulatory considerations on artificial intelligence for health	2023	World Health Organization (WHO)	International regulatory guidance / policy report	Global	AI; Health; Ethics; Regulations	Human Oversight and Clinical Responsibility	institutional and regulatory responsibility; Accountability; human oversight;	Responsibility; Beneficence; Non-maleficence	The report emphasises that regulatory frameworks must ensure that final clinical responsibility remains with qualified human professionals and that AI systems are subject to continuous oversight throughout the total product lifecycle	Requirement for human-in-the-loop or human-on-the-loop mechanisms and clear attribution of responsibility for AI-supported decisions	Ensures that AI-supported rehabilitation or exercise recommendations are always supervised and validated by healthcare professionals in vulnerable palliative care contexts.	guideline 1 and 3 - AI should complement—and never replace—human clinical judgement.. There should be constant clinical supervision of the exercises suggested by AI.	Sections 5.2, 6	https://www.who.int/publications/i/item/9789240074897
Regulatory considerations on artificial intelligence for health	2023	World Health Organization (WHO)	International regulatory guidance / policy report	Global	AI; Health; Ethics; Regulations	Patient Autonomy, Preferences, and Proportionality	professional responsability; accountability mechanisms	Autonomy & Consent; Dignity; Fairness & non-discrimination	WHO highlights the importance of aligning AI systems with their intended medical purpose and applying proportional regulatory requirements based on risk, ensuring that deployment does not expose patients to disproportionate harm	Obligation to ensure informed consent, respect for personal values, and safeguards for individuals with reduced decision-making capacity. Risk-based regulatory approach; pre-specification of intended medical purpose; proportionality between AI system risk and regulatory requirements; consideration of context and population in which AI is deployed.	Supports person-centred rehabilitation decisions aligned with patient goals of care and limits the use of AI-driven interventions that may be disproportionate. Definition and documentation of intended use; limitation of AI deployment to contexts where benefits outweigh potential burdens; ongoing reassessment of appropriateness of AI-supported interventions, particularly in vulnerable or high-risk populations.	guideline 2 and 5- AI recommendations should be tailored to the patient's preferences and limitations. The informed consent of the patient and/or carer must be obtained.	Sections 5.1.2, 5.1.4	https://www.who.int/publications/i/item/9789240074897
Regulatory considerations on artificial intelligence for health	2023	World Health Organization (WHO)	International regulatory guidance / policy report	Global	AI; Health; Ethics; Regulations	Transparency and Explainability	auditability; risk classification	Transparency & explainability; Safety & robustness; Accountability	Documentation and transparency are identified as foundational regulatory requirements to enable trust, traceability, and regulatory oversight of AI systems used in health	Expectation that AI systems used in healthcare provide understandable explanations and support auditability and traceability. Mandatory documentation across the total AI lifecycle; traceability of development decisions; transparency regarding data sources, model updates, and validation processes; documentation proportional to system risk.	Allows clinicians to understand and explain AI-supported rehabilitation recommendations to patients and caregivers, fostering trust and informed decision-making. Clear documentation of datasets, model design, validation, and updates; availability of information to regulators and end-users; traceable records enabling audit, regulatory review, and clinical accountability.	guideline 4 and 7- The choice of technology should be based on validated and transparent evidence; The use of AI should be transparent and shared with the entire interdisciplinary team.	Topic Area 1; Section 5.1	https://www.who.int/publications/i/item/9789240074897
Regulatory considerations on artificial intelligence for health	2023	World Health Organization (WHO)	International regulatory guidance / policy report	Global	AI; Health; Ethics; Regulations	Fairness, Equity, and Non-Discrimination	oversight requirements; accountability mechanisms	Fairness & non-discrimination; justice; protection of vulnerable populations	The report explicitly warns that non-representative datasets and poor data quality can introduce bias and inequity, particularly affecting vulnerable populations, and calls for proactive bias mitigation and monitoring	Institutional and regulatory obligation to prevent algorithmic bias and ensure that AI systems do not exacerbate existing health inequalities through biased or incomplete datasets.	Highly relevant to palliative care rehabilitation, where patients are frequently underrepresented in datasets and at increased risk of exclusion from data-driven decision-making.	guideline 6-The algorithms used must respect the principles of fairness and equity in access.	Sections 5.4.2–5.4.3	https://www.who.int/publications/i/item/9789240074897
Regulatory considerations on artificial intelligence for health	2023	World Health Organization (WHO)	International regulatory guidance / policy report	Global	AI; Health; Ethics; Regulations	Professional Competence and Ethical Literacy	professional responsability; accountability mechanisms	Professional responsibility; transparency & explainability	WHO stresses that effective regulation and safe deployment of AI require appropriately trained users, organisational responsibility, and ongoing engagement and collaboration among stakeholders	Institutional responsibility to ensure adequate training and ethical competence for professionals using AI in healthcare.	Supports the need for trained rehabilitation professionals capable of critically and ethically using AI tools in sensitive palliative care settings.	guideline 8- The training of professionals in digital literacy and algorithmic ethics must be ongoing.	Topic Area 6; Sections 5.6, 6	https://www.who.int/publications/i/item/9789240074897
Recommendation of the Council on Artificial Intelligence	2019	OECD	International policy and regulatory recommendation	Global (OECD member and partner countries)	AI; governance; accountability; healthcare; policy	Human Oversight and Clinical Responsibility	Accountability mechanisms; human control; allocation of responsibility	Responsibility; Beneficence; Non-maleficence; Accountability	"AI actors should be accountable for the proper functioning of AI systems and for the respect of the above principles, based on their roles, the context, and their ability to act."	Requirement for human-in-the-loop or human-on-the-loop mechanisms and clear attribution of responsibility for AI-supported decisions	Ensures that AI-supported rehabilitation or exercise recommendations are always supervised and validated by healthcare professionals in vulnerable palliative care contexts.	guideline 1 and 3 - AI should complement—and never replace—human clinical judgement.. There should be constant clinical supervision of the exercises suggested by AI.	OECD frames accountability as context-dependent, reinforcing relevance for high-vulnerability clinical settings.	https://legalinstruments.oecd.org/en/instruments/OECD-LEGAL-0449
Recommendation of the Council on Artificial Intelligence	2019	OECD	International policy and regulatory recommendation	Global (OECD member and partner countries)	AI; governance; accountability; healthcare; policy	Patient Autonomy, Preferences, and Proportionality	Risk-based approach; respect for human-centred values	Autonomy; Dignity; Human-centred values	"AI systems should be designed in a manner that respects the rule of law, human rights, democratic values and diversity, and they should include appropriate safeguards."	Obligation to ensure informed consent, respect for personal values, and safeguards for individuals with reduced decision-making capacity. Risk-based regulatory approach; pre-specification of intended medical purpose; proportionality between AI system risk and regulatory requirements; consideration of context and population in which AI is deployed.	Supports person-centred rehabilitation decisions aligned with patient goals of care and limits the use of AI-driven interventions that may be disproportionate. Definition and documentation of intended use; limitation of AI deployment to contexts where benefits outweigh potential burdens; ongoing reassessment of appropriateness of AI-supported interventions, particularly in vulnerable or high-risk populations.	guideline 2 and 5- AI recommendations should be tailored to the patient's preferences and limitations. The informed consent of the patient and/or carer must be obtained.	OECD does not use "palliative" language, but human-centred framing is directly transferable.	https://legalinstruments.oecd.org/en/instruments/OECD-LEGAL-0449

Recommendation of the Council on Artificial Intelligence	2019	OECD	International policy and regulatory recommendation	Global (OECD member and partner countries)	AI; governance; accountability; healthcare; policy	Transparency and Explainability	Transparency; traceability; explainability	Transparency; Accountability; Trust	"AI actors should commit to transparency and responsible disclosure regarding AI systems."	Expectation that AI systems used in healthcare provide understandable explanations and support auditability and traceability. Mandatory documentation across the total AI lifecycle; traceability of development decisions; transparency regarding data sources, model updates, and validation processes; documentation proportional to system risk.	Allows clinicians to understand and explain AI-supported rehabilitation recommendations to patients and caregivers, fostering trust and informed decision-making. Clear documentation of datasets, model design, validation, and updates; availability of information to regulators and end-users; traceable records enabling audit, regulatory review, and clinical accountability.	guideline 4 and 7- The choice of technology should be based on validated and transparent evidence; The use of AI should be transparent and shared with the entire interdisciplinary team.	OECD transparency principle underpins later WHO and EU regulatory requirements.	https://legalinstruments.oecd.org/en/instruments/OECD-LEGAL-0449
Recommendation of the Council on Artificial Intelligence	2019	OECD	International policy and regulatory recommendation	Global (OECD member and partner countries)	AI; governance; accountability; healthcare; policy	Fairness, Equity, and Non-Discrimination	Inclusive growth; non-discrimination; monitoring of impacts	Justice; Equity; Non-discrimination	"AI actors should promote inclusive growth, sustainable development and well-being."	Institutional and regulatory obligation to prevent algorithmic bias and ensure that AI systems do not exacerbate existing health inequalities through biased or incomplete datasets.	Highly relevant to palliative care rehabilitation, where patients are frequently underrepresented in datasets and at increased risk of exclusion from data-driven decision-making.	guideline 6-The algorithms used must respect the principles of fairness and equity in access.	OECD explicitly links fairness to societal-level impacts, strengthening regulatory relevance.	https://legalinstruments.oecd.org/en/instruments/OECD-LEGAL-0449
Recommendation of the Council on Artificial Intelligence	2019	OECD	International policy and regulatory recommendation	Global (OECD member and partner countries)	AI; governance; accountability; healthcare; policy	Professional Competence and Ethical Literacy	Capacity building; workforce preparedness; institutional responsibility	Professional responsibility; Integrity; Accountability	"Governments should invest in AI research and development, foster a digital ecosystem for AI, and build human capacity."	Institutional responsibility to ensure adequate training and ethical competence for professionals using AI in healthcare.	Supports the need for trained rehabilitation professionals capable of critically and ethically using AI tools in sensitive palliative care settings.	guideline 8- The training of professionals in digital literacy and algorithmic ethics must be ongoing.		https://legalinstruments.oecd.org/en/instruments/OECD-LEGAL-0449
Ethics Guidelines for Trustworthy Artificial Intelligence	2019	High-Level Expert Group on Artificial Intelligence (European Commission)	Ethical guideline / policy framework	EU	Trustworthy AI; ethics; governance; healthcare	Human Oversight and Clinical Responsibility	Human oversight requirements; accountability mechanisms; governance structures	Responsibility; Beneficence; Non-maleficence; Accountability	"AI systems should support human autonomy and decision-making, as prescribed by the principle of respect for human autonomy." The guidelines emphasise human-in-the-loop, human-on-the-loop and human-in-command approaches.	Requirement for human-in-the-loop or human-on-the-loop mechanisms and clear attribution of responsibility for AI-supported decisions	Ensures that AI-supported rehabilitation or exercise recommendations are always supervised and validated by healthcare professionals in vulnerable palliative care contexts.	guideline 1 and 3 - AI should complement—and never replace—human clinical judgement.. There should be constant clinical supervision of the exercises suggested by AI.	Introduces graded models of human oversight later reflected in the EU AI Act.	https://digital-strategy.ec.europa.eu/en/library/ethics-guidelines-trustworthy-ai
Ethics Guidelines for Trustworthy Artificial Intelligence	2019	High-Level Expert Group on Artificial Intelligence (European Commission)	Ethical guideline / policy framework	EU	Trustworthy AI; ethics; governance; healthcare	Patient Autonomy, Preferences, and Proportionality	Human-centred design; proportionality; respect for fundamental rights	Autonomy; Dignity; Human-centred values	"Trustworthy AI should respect fundamental rights, including human autonomy, and should not diminish or mislead human decision-making."	Obligation to ensure informed consent, respect for personal values, and safeguards for individuals with reduced decision-making capacity. Risk-based regulatory approach; pre-specification of intended medical purpose; proportionality between AI system risk and regulatory requirements; consideration of context and population in which AI is deployed.	Supports person-centred rehabilitation decisions aligned with patient goals of care and limits the use of AI-driven interventions that may be disproportionate. Definition and documentation of intended use; limitation of AI deployment to contexts where benefits outweigh potential burdens; ongoing reassessment of appropriateness of AI-supported interventions, particularly in vulnerable or high-risk populations.	guideline 2 and 5- AI recommendations should be tailored to the patient's preferences and limitations. The informed consent of the patient and/or carer must be obtained.	Strong alignment with palliative care values despite absence of explicit palliative reference.	https://digital-strategy.ec.europa.eu/en/library/ethics-guidelines-trustworthy-ai
Ethics Guidelines for Trustworthy Artificial Intelligence	2019	High-Level Expert Group on Artificial Intelligence (European Commission)	Ethical guideline / policy framework	EU	Trustworthy AI; ethics; governance; healthcare	Transparency and Explainability	Traceability; explainability; communication obligations	Transparency; Accountability; Trust	"The data, system and AI business models should be transparent. Traceability mechanisms can help achieve this."	Expectation that AI systems used in healthcare provide understandable explanations and support auditability and traceability. Mandatory documentation across the total AI lifecycle; traceability of development decisions; transparency regarding data sources, model updates, and validation processes; documentation proportional to system risk.	Allows clinicians to understand and explain AI-supported rehabilitation recommendations to patients and caregivers, fostering trust and informed decision-making. Clear documentation of datasets, model design, validation, and updates; availability of information to regulators and end-users; traceable records enabling audit, regulatory review, and clinical accountability.	guideline 4 and 7- The choice of technology should be based on validated and transparent evidence; The use of AI should be transparent and shared with the entire interdisciplinary team.	Introduces the concept of traceability later embedded in regulatory requirements.	https://digital-strategy.ec.europa.eu/en/library/ethics-guidelines-trustworthy-ai
Ethics Guidelines for Trustworthy Artificial Intelligence	2019	High-Level Expert Group on Artificial Intelligence (European Commission)	Ethical guideline / policy framework	EU	Trustworthy AI; ethics; governance; healthcare	Fairness, Equity, and Non-Discrimination	Bias mitigation; inclusiveness; monitoring of discriminatory outcomes	Justice; Equity; Non-discrimination	"AI systems should be inclusive and accessible, and should not lead to unfair bias or discrimination."	Institutional and regulatory obligation to prevent algorithmic bias and ensure that AI systems do not exacerbate existing health inequalities through biased or incomplete datasets.	Highly relevant to palliative care rehabilitation, where patients are frequently underrepresented in datasets and at increased risk of exclusion from data-driven decision-making.	guideline 6-The algorithms used must respect the principles of fairness and equity in access.	Explicitly identifies vulnerable groups as at higher risk of harm.	https://digital-strategy.ec.europa.eu/en/library/ethics-guidelines-trustworthy-ai
Ethics Guidelines for Trustworthy Artificial Intelligence	2019	High-Level Expert Group on Artificial Intelligence (European Commission)	Ethical guideline / policy framework	EU	Trustworthy AI; ethics; governance; healthcare	Professional Competence and Ethical Literacy	Training obligations; organisational preparedness; governance culture	Professional responsibility; Integrity; Accountability	"Those developing, deploying or using AI systems should be trained and informed about the system's capabilities and limitations."	Institutional responsibility to ensure adequate training and ethical competence for professionals using AI in healthcare.	Supports the need for trained rehabilitation professionals capable of critically and ethically using AI tools in sensitive palliative care settings.	guideline 8- The training of professionals in digital literacy and algorithmic ethics must be ongoing.	Strong bridge between ethics and governance implementation.	https://digital-strategy.ec.europa.eu/en/library/ethics-guidelines-trustworthy-ai
Ethics and Governance of Artificial Intelligence for Health	2021	World Health Organization (WHO)	International ethical and governance guideline	Global	AI; health; ethics; governance; regulation	Human Oversight and Clinical Responsibility	Human oversight requirements; accountability mechanisms; governance across AI lifecycle	Responsibility; Beneficence; Non-maleficence; Accountability	WHO emphasises that AI systems for health must not replace human decision-making and that accountability for outcomes must remain with human actors throughout the AI lifecycle.	Requirement for human-in-the-loop or human-on-the-loop mechanisms and clear attribution of responsibility for AI-supported decisions	Ensures that AI-supported rehabilitation or exercise recommendations are always supervised and validated by healthcare professionals in vulnerable palliative care contexts.	guideline 1 and 3 - AI should complement—and never replace—human clinical judgement.. There should be constant clinical supervision of the exercises suggested by AI.	Strong emphasis on accountability and governance responsibilities of institutions and professionals.	https://www.who.int/publications/i/item/9789240029200
Ethics and Governance of Artificial Intelligence for Health	2021	World Health Organization (WHO)	International ethical and governance guideline	Global	AI; health; ethics; governance; regulation	Patient Autonomy, Preferences, and Proportionality	Protection of human rights; informed consent; proportionality; safeguards for vulnerable populations	Autonomy; Dignity; Respect for persons; Proportionality	WHO states that AI for health should respect autonomy, informed consent, and human rights, particularly when deployed in contexts involving vulnerable populations.	Obligation to ensure informed consent, respect for personal values, and safeguards for individuals with reduced decision-making capacity. Risk-based regulatory approach; pre-specification of intended medical purpose; proportionality between AI system risk and regulatory requirements; consideration of context and population in which AI is deployed.	Supports person-centred rehabilitation decisions aligned with patient goals of care and limits the use of AI-driven interventions that may be disproportionate. Definition and documentation of intended use; limitation of AI deployment to contexts where benefits outweigh potential burdens; ongoing reassessment of appropriateness of AI-supported interventions, particularly in vulnerable or high-risk populations.	guideline 2 and 5- AI recommendations should be tailored to the patient's preferences and limitations. The informed consent of the patient and/or carer must be obtained.	Explicit recognition of vulnerability strengthens relevance to palliative care.	https://www.who.int/publications/i/item/9789240029200
Ethics and Governance of Artificial Intelligence for Health	2021	World Health Organization (WHO)	International ethical and governance guideline	Global	AI; health; ethics; governance; regulation	Transparency and Explainability	Explainability requirements; transparency obligations; documentation and traceability	Transparency; Accountability; Trust	The WHO framework highlights transparency and explainability as prerequisites for trust, accountability, and safe integration of AI into health systems.	Expectation that AI systems used in healthcare provide understandable explanations and support auditability and traceability. Mandatory documentation across the total AI lifecycle; traceability of development decisions; transparency regarding data sources, model updates, and validation processes; documentation proportional to system risk.	Allows clinicians to understand and explain AI-supported rehabilitation recommendations to patients and caregivers, fostering trust and informed decision-making. Clear documentation of datasets, model design, validation, and updates; availability of information to regulators and end-users; traceable records enabling audit, regulatory review, and clinical accountability.	guideline 4 and 7- The choice of technology should be based on validated and transparent evidence; The use of AI should be transparent and shared with the entire interdisciplinary team.	Transparency framed as both ethical and governance requirement.	https://www.who.int/publications/i/item/9789240029200

Ethics and Governance of Artificial Intelligence for Health	2021	World Health Organization (WHO)	International ethical and governance guideline	Global	AI; health; ethics; governance; regulation	Fairness, Equity, and Non-Discrimination	Bias mitigation; inclusive design; equity monitoring	Justice; Equity; Non-discrimination	WHO warns that biased data and inequitable deployment of AI systems can exacerbate health inequalities and disproportionately harm marginalised populations.	Institutional and regulatory obligation to prevent algorithmic bias and ensure that AI systems do not exacerbate existing health inequalities through biased or incomplete datasets.	Highly relevant to palliative care rehabilitation, where patients are frequently underrepresented in datasets and at increased risk of exclusion from data-driven decision-making.	guideline 6-The algorithms used must respect the principles of fairness and equity in access.	Strong alignment with WHO's health equity mandate.	https://www.who.int/publications/i/item/9789240029200
Ethics and Governance of Artificial Intelligence for Health	2021	World Health Organization (WHO)	International ethical and governance guideline	Global	AI; health; ethics; governance; regulation	Professional Competence and Ethical Literacy	Capacity building; professional training; institutional responsibility	Professional responsibility; Ethical competence; Accountability	WHO highlights the need for education, training, and organisational capacity to ensure the ethical and safe use of AI for health.	Institutional responsibility to ensure adequate training and ethical competence for professionals using AI in healthcare.	Supports the need for trained rehabilitation professionals capable of critically and ethically using AI tools in sensitive palliative care settings.	guideline 8- The training of professionals in digital literacy and algorithmic ethics must be ongoing.	Emphasises shared responsibility between institutions and professionals.	https://www.who.int/publications/i/item/9789240029200
White Paper on Artificial Intelligence: A European approach to excellence and trust	2020	European Commission	Strategy paper / policy framework	EU	AI; regulation; trust; healthcare	Human Oversight and Clinical Responsibility	Human oversight requirements; accountability mechanisms; risk-based governance; allocation of responsibility	Responsibility; Beneficence; Non-maleficence; Accountability	The White Paper stresses that high-risk AI systems must be subject to appropriate human oversight to prevent or minimise risks and ensure accountability, particularly in sensitive sectors such as healthcare.	Requirement for human-in-the-loop or human-on-the-loop mechanisms and clear attribution of responsibility for AI-supported decisions	Ensures that AI-supported rehabilitation or exercise recommendations are always supervised and validated by healthcare professionals in vulnerable palliative care contexts.	guideline 1 and 3 - AI should complement—and never replace—human clinical judgement.. There should be constant clinical supervision of the exercises suggested by AI.	Introduces human oversight as a cornerstone of "trustworthy AI" later embedded in the EU AI Act.	https://eur-lex.europa.eu/legal-content/EN/TXT/?uri=CELEX:52020DC0065
White Paper on Artificial Intelligence: A European approach to excellence and trust	2020	European Commission	Strategy paper / policy framework	EU	AI; regulation; trust; healthcare	Patient Autonomy, Preferences, and Proportionality	Risk-based approach; proportional safeguards; protection of fundamental rights	Autonomy; Dignity; Proportionality; Human rights	The Commission emphasises that AI regulation should be proportionate to the level of risk and should ensure protection of fundamental rights, particularly in high-risk applications affecting individuals.	Obligation to ensure informed consent, respect for personal values, and safeguards for individuals with reduced decision-making capacity. Risk-based regulatory approach; pre-specification of intended medical purpose; proportionality between AI system risk and regulatory requirements; consideration of context and population in which AI is deployed.	Supports person-centred rehabilitation decisions aligned with patient goals of care and limits the use of AI-driven interventions that may be disproportionate. Definition and documentation of intended use; limitation of AI deployment to contexts where benefits outweigh potential burdens; ongoing reassessment of appropriateness of AI-supported interventions, particularly in vulnerable or high-risk populations.	guideline 2 and 5- AI recommendations should be tailored to the patient's preferences and limitations. The informed consent of the patient and/or carer must be obtained.	Although not palliative-specific, the proportionality logic is directly transferable to palliative contexts.	https://eur-lex.europa.eu/legal-content/EN/TXT/?uri=CELEX:52020DC0065
White Paper on Artificial Intelligence: A European approach to excellence and trust	2020	European Commission	Strategy paper / policy framework	EU	AI; regulation; trust; healthcare	Transparency and Explainability	Transparency obligations; traceability; information duties for high-risk AI systems	Accountability; Trust	The White Paper identifies transparency and traceability as essential requirements for trustworthy AI, particularly for high-risk applications such as healthcare.	Expectation that AI systems used in healthcare provide understandable explanations and support auditability and traceability. Mandatory documentation across the total AI lifecycle; traceability of development decisions; transparency regarding data sources, model updates, and validation processes; documentation proportional to system risk.	Allows clinicians to understand and explain AI-supported rehabilitation recommendations to patients and caregivers, fostering trust and informed decision-making. Clear documentation of datasets, model design, validation, and updates; availability of information to regulators and end-users; traceable records enabling audit, regulatory review, and clinical accountability.	guideline 4 and 7- The choice of technology should be based on validated and transparent evidence; The use of AI should be transparent and shared with the entire interdisciplinary team.	Transparency framed as both a user right and a regulatory control mechanism.	https://eur-lex.europa.eu/legal-content/EN/TXT/?uri=CELEX:52020DC0065
White Paper on Artificial Intelligence: A European approach to excellence and trust	2020	European Commission	Strategy paper / policy framework	EU	AI; regulation; trust; healthcare	Fairness, Equity, and Non-Discrimination	Bias prevention; representative data requirements; non-discrimination safeguards	Justice; Equity; Non-discrimination	The Commission warns that AI systems can perpetuate or amplify bias and discrimination if not properly designed and governed, particularly in high-risk sectors.	Institutional and regulatory obligation to prevent algorithmic bias and ensure that AI systems do not exacerbate existing health inequalities through biased or incomplete datasets.	Highly relevant to palliative care rehabilitation, where patients are frequently underrepresented in datasets and at increased risk of exclusion from data-driven decision-making.	guideline 6-The algorithms used must respect the principles of fairness and equity in access.	Explicit focus on vulnerable groups strengthens applicability to palliative care.	https://eur-lex.europa.eu/legal-content/EN/TXT/?uri=CELEX:52020DC0065
White Paper on Artificial Intelligence: A European approach to excellence and trust	2020	European Commission	Strategy paper / policy framework	EU	AI; regulation; trust; healthcare	Professional Competence and Ethical Literacy	Training obligations; organisational responsibility; governance culture	Professional responsibility; Integrity; Accountability	The White Paper highlights the need for skills development, training, and organisational preparedness to ensure the responsible use of AI systems.	Institutional responsibility to ensure adequate training and ethical competence for professionals using AI in healthcare.	Supports the need for trained rehabilitation professionals capable of critically and ethically using AI tools in sensitive palliative care settings.	guideline 8- The training of professionals in digital literacy and algorithmic ethics must be ongoing.	Strong link between governance effectiveness and workforce competence.	https://eur-lex.europa.eu/legal-content/EN/TXT/?uri=CELEX:52020DC0065
Artificial Intelligence Act (AI Act)	2024	European Union (European Parliament & Council)	Binding regulatory framework (EU Regulation)	EU	AI Act; regulation; high-risk AI; healthcare;	Human Oversight and Clinical Responsibility	Mandatory human oversight; allocation of responsibility; governance of high-risk AI systems	Responsibility; Beneficence; Non-maleficence; Accountability	The AI Act requires that high-risk AI systems be designed and developed to allow for effective human oversight, ensuring that natural persons can understand, monitor, and intervene in system operation where necessary.	Requirement for human-in-the-loop or human-on-the-loop mechanisms and clear attribution of responsibility for AI-supported decisions	Ensures that AI-supported rehabilitation or exercise recommendations are always supervised and validated by healthcare professionals in vulnerable palliative care contexts.	guideline 1 and 3 - AI should complement—and never replace—human clinical judgement.. There should be constant clinical supervision of the exercises suggested by AI.	Healthcare AI systems are explicitly classified as high-risk. Political agreement / final adopted text	https://artificialintelligenceact.eu
Artificial Intelligence Act (AI Act)	2024	European Union (European Parliament & Council)	Binding regulatory framework (EU Regulation)	EU	AI Act; regulation; high-risk AI; healthcare;	Patient Autonomy, Preferences, and Proportionality	Risk-based classification; proportional safeguards; protection of fundamental rights	Autonomy; Dignity; Proportionality; Fundamental rights	The AI Act adopts a risk-based regulatory approach, requiring stricter obligations for AI systems that pose higher risks to fundamental rights, health, and safety.	Obligation to ensure informed consent, respect for personal values, and safeguards for individuals with reduced decision-making capacity. Risk-based regulatory approach; pre-specification of intended medical purpose; proportionality between AI system risk and regulatory requirements; consideration of context and population in which AI is deployed.	Supports person-centred rehabilitation decisions aligned with patient goals of care and limits the use of AI-driven interventions that may be disproportionate. Definition and documentation of intended use; limitation of AI deployment to contexts where benefits outweigh potential burdens; ongoing reassessment of appropriateness of AI-supported interventions, particularly in vulnerable or high-risk populations.	guideline 2 and 5- AI recommendations should be tailored to the patient's preferences and limitations. The informed consent of the patient and/or carer must be obtained.	Proportionality logic aligns strongly with palliative care ethics.	https://artificialintelligenceact.eu
Artificial Intelligence Act (AI Act)	2024	European Union (European Parliament & Council)	Binding regulatory framework (EU Regulation)	EU	AI Act; regulation; high-risk AI; healthcare;	Transparency and Explainability	Transparency obligations; user information duties; traceability and documentation requirements	Transparency; Accountability; Trust	The AI Act establishes transparency obligations for high-risk AI systems, including requirements to provide users with information necessary to interpret system outputs and use them appropriately.	Expectation that AI systems used in healthcare provide understandable explanations and support auditability and traceability. Mandatory documentation across the total AI lifecycle; traceability of development decisions; transparency regarding data sources, model updates, and validation processes; documentation	Allows clinicians to understand and explain AI-supported rehabilitation recommendations to patients and caregivers, fostering trust and informed decision-making. Clear documentation of datasets, model design, validation, and updates; availability of information to regulators and end-users; traceable records enabling audit, regulatory review, and clinical accountability.	guideline 4 and 7- The choice of technology should be based on validated and transparent evidence; The use of AI should be transparent and shared with the entire interdisciplinary team.	Transparency is framed as both a user right and a compliance obligation.	https://artificialintelligenceact.eu

Artificial Intelligence Act (AI Act)	2024	European Union (European Parliament & Council)	Binding regulatory framework (EU Regulation)	EU	AI Act; regulation; high-risk AI; healthcare;	Fairness, Equity, and Non-Discrimination	Bias mitigation; data governance; monitoring of discriminatory outcomes	Justice; Equity; Non-discrimination	The AI Act requires that training, validation, and testing datasets for high-risk AI systems be relevant, representative, and sufficiently complete to minimise risks of bias and discrimination.	Institutional and regulatory obligation to prevent algorithmic bias and ensure that AI systems do not exacerbate existing health inequalities through biased or incomplete datasets.	Highly relevant to palliative care rehabilitation, where patients are frequently underrepresented in datasets and at increased risk of exclusion from data-driven decision-making.	guideline 6-The algorithms used must respect the principles of fairness and equity in access.	One of the strongest legally enforceable fairness provisions in global AI regulation.	https://artificialintelligenceact.eu
Artificial Intelligence Act (AI Act)	2024	European Union (European Parliament & Council)	Binding regulatory framework (EU Regulation)	EU	AI Act; regulation; high-risk AI; healthcare;	Professional Competence and Ethical Literacy	User training obligations; organisational responsibility; compliance culture	Professional responsibility; Accountability; Safety	The AI Act requires that users of high-risk AI systems receive adequate instructions and that organisations ensure appropriate human oversight and competence in system use.	Institutional responsibility to ensure adequate training and ethical competence for professionals using AI in healthcare.	Supports the need for trained rehabilitation professionals capable of critically and ethically using AI tools in sensitive palliative care settings.	guideline 8- The training of professionals in digital literacy and algorithmic ethics must be ongoing.	Moves ethical competence from “good practice” to regulatory expectation.	https://artificialintelligenceact.eu

This supplementary table presents the documentary analysis and data extraction process underpinning the identification of ethical–regulatory domains.

The table summarises how international ethical frameworks, policy documents, and regulatory instruments were analysed and mapped to convergent domains relevant to the use of artificial intelligence in palliative care rehabilitation.
