# Supplementary material for: Ethical–Regulatory Guidelines for AI in Palliative Care Rehabilitation
Source: Healthcare (Basel). 2026 Mar 31;14(7):895. doi: 10.3390/healthcare14070895 (PMC13072931; doi:10.3390/healthcare14070895)
Supplement: Supplementary file 1 [file healthcare-14-00895-s001.zip › Supplementary_Material_S1_Institutional_Documents.pdf]

Ethical regulatory guidelines for AI in palliative care rehabilitation

Supplementary Material S1

Institutional Documentary Corpus

The following authoritative institutional documents constituted the primary documentary corpus analysed in this study. These documents were identified through purposive documentary identification based on the inclusion criteria described in Section 2.2 of the Methods.

The corpus includes international ethical frameworks, governance guidelines, policy reports, and regulatory instruments addressing ethical, governance, and regulatory aspects of artificial intelligence in healthcare.

All documents were accessed through official institutional websites between January 1st and January 30th, 2026. URLs correspond to the official sources from which the documents were retrieved.

1. UNESCO

UNESCO. Recommendation on the Ethics of Artificial Intelligence; United Nations Educational, Scientific and Cultural Organization: Paris, France, 2021.
Available online: Recommendation on the Ethics of Artificial Intelligence | UNESCO
Accessed on: January 2026.

2. EPRS

European Parliamentary Research Service (EPRS). Artificial Intelligence in Healthcare: Applications, Risks, and Ethical and Societal Impacts; European Parliament: Brussels, Belgium, 2022.
Available online: https://www.europarl.europa.eu/thinktank
Accessed on: January 2026.

3. EU Health Inequalities Statement

EU Health Policy Platform. The Impact of Artificial Intelligence on Health Outcomes for Key Populations: Navigating Health Inequalities in the EU – Final Joint Statement; European Commission: Brussels, Belgium, 2023.
Available online: https://health.ec.europa.eu
Accessed on: January 2026.

Ethical regulatory guidelines for AI in palliative care rehabilitation

4. WHO 2023

World Health Organization (WHO). Regulatory Considerations on Artificial Intelligence for Health; WHO: Geneva, Switzerland, 2023.
Available online: https://www.who.int/publications/i/item/9789240074897
Accessed on: January 2026.

5. OECD

Organisation for Economic Co-operation and Development (OECD). Recommendation of the Council on Artificial Intelligence; OECD: Paris, France, 2019.
Available online: https://legalinstruments.oecd.org/en/instruments/OECD-LEGAL-0449
Accessed on: January 2026.

6. EU Ethics Guidelines

High-Level Expert Group on Artificial Intelligence. Ethics Guidelines for Trustworthy Artificial Intelligence; European Commission: Brussels, Belgium, 2019.
Available online: https://digital-strategy.ec.europa.eu/en/library/ethics-guidelines-trustworthy-ai
Accessed on: January 2026.

7. WHO 2021

World Health Organization (WHO). Ethics and Governance of Artificial Intelligence for Health; WHO: Geneva, Switzerland, 2021.
Available online: https://www.who.int/publications/i/item/9789240029200
Accessed on: January 2026.

8. EU White Paper

European Commission. White Paper on Artificial Intelligence: A European Approach to Excellence and Trust; European Commission: Brussels, Belgium, 2020.
Available online: https://eur-lex.europa.eu/legal-content/EN/TXT/?uri=CELEX:52020DC0065
Accessed on: January 2026.

9. AI Act

European Union. Regulation (EU) 2024/1689 of the European Parliament and of the Council Laying Down Harmonised Rules on Artificial Intelligence (Artificial Intelligence Act); European Union: Brussels, Belgium, 2024.
Available online: https://artificialintelligenceact.eu
Accessed on: January 2026.

This supplementary material lists the institutional policy, governance, and regulatory documents that constituted the primary documentary corpus analysed in this study.
