# Supplementary material for: Ethical–Regulatory Guidelines for AI in Palliative Care Rehabilitation
Source: Healthcare (Basel). 2026 Mar 31;14(7):895. doi: 10.3390/healthcare14070895 (PMC13072931; doi:10.3390/healthcare14070895)
Supplement: Supplementary file 1 [file healthcare-14-00895-s001.zip › Supplementary_Material_S2_Academic_Search_Strategy.pdf]

# Ethical regulatory guidelines for AI in palliative care rehabilitation

## Supplementary Material S2

## Academic Literature Search Strategy

To support conceptual contextualisation of ethical–regulatory themes identified in the documentary corpus, exploratory database searches were conducted in January 2026 in PubMed/MEDLINE, Scopus, and Web of Science.

The purpose of these searches was to identify academic discussions addressing the intersection of artificial intelligence, ethics, governance, rehabilitation, and palliative care. The searches were not designed as systematic reviews and did not constitute part of the primary documentary dataset analysed in this study.

Rather, they served to support conceptual alignment and to verify the absence of integrated ethical–regulatory frameworks specific to AI-supported rehabilitation in palliative care contexts.

#### 1. PubMed/MEDLINE

(("Artificial Intelligence"[Mesh] OR "artificial intelligence"[Title/Abstract] OR "machine learning"[Title/Abstract] OR "algorithm*"[Title/Abstract]) AND ("Ethics"[Mesh] OR "ethics"[Title/Abstract] OR "bioethics"[Title/Abstract] OR "governance"[Title/Abstract] OR "regulation"[Title/Abstract] OR "regulatory"[Title/Abstract]) AND ("Rehabilitation"[Mesh] OR "rehabilitation"[Title/Abstract] OR "physical therapy"[Title/Abstract] OR "physiotherapy"[Title/Abstract]) AND ("Palliative Care"[Mesh] OR "palliative care"[Title/Abstract] OR "end-of-life care"[Title/Abstract]))

#### 2. Scopus

(TITLE-ABS-KEY("artificial intelligence" OR "machine learning" OR algorithm*) AND TITLE-ABS-KEY(ethics OR bioethics OR governance OR regulation OR regulatory) AND TITLE-ABS-KEY(rehabilitation OR "physical therapy" OR physiotherapy) AND TITLE-ABS-KEY("palliative care" OR "end-of-life care"))

#### 3. Web of Science (Core Collection)

(TS=("artificial intelligence" OR "machine learning" OR algorithm*) AND TS=(ethics OR bioethics OR governance OR regulation OR regulatory) AND TS=(rehabilitation OR "physical therapy" OR physiotherapy) AND TS=("palliative care" OR "end-of-life care"))

#### Methodological Note

No formal screening protocol, quality appraisal or systematic synthesis procedures were applied, as the academic literature was consulted solely for contextual grounding rather than domain generation or empirical evidence aggregation.
